# Towards harmonization of directly measured free 25-hydroxyvitamin D using an enzyme-linked immunosorbent assay

**DOI:** 10.1007/s00216-022-04313-y

**Published:** 2022-09-16

**Authors:** Christopher T. Sempos, Ernst Lindhout, Nicolas Heureux, Michel Hars, Damon A. Parkington, Emily Dennison, Ramón Durazo-Arvizu, Kerry S. Jones, Stephen A. Wise

**Affiliations:** 1 Office of Dietary Supplements, National Institutes of Health, Bethesda, MD 20892 USA; 2 Vitamin D Standardization Program, LLC, Havre de Grace, MD 21078 USA; 3 Future Diagnostics, Nieuweweg 279, 6603 BN Wijchen, Netherlands; 4 DIAsource Immunoassays, B-1348 Louvain-La-Neuve, Belgium; 5 BioSweet srl, B-5030 Lonzee, Belgium; 6 Nutritional Biomarker Laboratory, MRC Epidemiology Unit, University of Cambridge, Cambridge, CB2 0AH UK; 7 Immuno-Biological Laboratories Inc (IBL-America), Minneapolis, MN 55432 USA; 8 The Saban Research Institute, Children’s Hospital Los Angeles, Los Angeles, CA 90027 USA

**Keywords:** Free 25-hydroxyvitamin D, Enzyme-linked immunosorbent assay (ELISA), Standard Reference Materials (SRMs), Total 25-hydroxyvitamin D, Vitamin D binding protein

## Abstract

The majority of circulating 25-hydroxyvitamin D (25(OH)D) is protein bound and perhaps less available than the free fraction of 25(OH)D; therefore, researchers have proposed that the measurement of free 25(OH)D in human serum may be a better indicator of vitamin D health status than total 25(OH)D. The availability of a new enzyme-linked immunosorbent assay (ELISA) for the determination of free 25(OH)D provides a method for direct measurement of the low levels of non-protein bound 25(OH)D. As an initial step towards harmonization of measurements of free 25(OH)D, the ELISA was used to measure free 25(OH)D in three existing Standard Reference Materials (SRMs): SRM 972a Vitamin D Metabolites in Frozen Human Serum, SRM 2973 Vitamin D Metabolites in Frozen Human Serum (High Level), and SRM 1949 Frozen Prenatal Human Serum. Target values for free 25(OH)D in the nine SRM serum pools, obtained by combining the results from two laboratories, ranged from 3.76 ± 0.36 to 10.0 ± 0.58 pg/mL. Of particular significance is the assignment of free 25(OH)D target values to SRM 1949, which consists of four serum pools from non-pregnant female donors of reproductive age and pregnant women in each of the three trimesters and which also has values assigned for vitamin D binding protein, which increases during pregnancy. The availability of target values for free 25(OH)D in these SRMs will allow researchers to validate new analytical methods and to compare their results with other researchers as an initial step towards harmonization of measurements among different studies and laboratories.

**Supplementary Information:**

The online version contains supplementary material available at 10.1007/s00216-022-04313-y.

## Introduction

Because of the importance of vitamin D in human health and development, in either the D_2_ (ergocalciferol) or D_3_ (cholecalciferol) form, testing for vitamin D in human serum has become routine to diagnose and monitor deficiency. The major circulating form of vitamin D and the primary marker of vitamin D status is 25-hydroxyvitamin D [25(OH)D], which is defined as the sum of 25-hydroxyvitamin D_2_ [25(OH)D_2_] and 25-hydroxyvitamin D_3_ [25(OH)D_3_]. Measurements of 25(OH)D are typically performed using a ligand binding assay, which responds to both 25(OH)D_2_ and 25(OH)D_3_ and thereby provides total 25(OH)D, or using liquid chromatography–tandem mass spectrometry (LC–MS/MS), which measures the 25(OH)D_2_ and 25(OH)D_3_ individually. Recent reviews [1–5] have discussed the challenges and difficulties in assessing vitamin D status, and recent studies [6–11] have demonstrated the variability of results among different 25(OH)D assays.

Like other steroid hormones, circulating 25(OH)D is primarily bound to proteins, with 85% to 90% strongly bound to vitamin D binding protein (VDBP), a specific transport protein for vitamin D; 10% to 15% is loosely bound to serum albumin; and only 0.02% to 0.04% exists in a free, unbound form [12–14]. Recent studies have suggested that the measurement of free 25(OH)D may be a better indicator of vitamin D status than the total 25(OH)D content [14–16], and several reviews have discussed the basis of these conclusions [12, 13, 15, 17, 18].

Methods for the determination of 25(OH)D have been available since the 1970s; however, methods for the determination of free 25(OH)D were first described in the 1980s based on centrifugal ultrafiltration using ^3^H-labeled 25(OH)D_3_ and ^14^C-labeled glucose. However, the method was not used extensively because it is time-consuming and expensive [19, 20]. Using affinity constants determined from centrifugal ultrafiltration measurements, Bikle et al. [19] proposed a calculation method in 1986 to determine free 25(OH)D using the measured total 25(OH)D, VDBP, and albumin concentrations and the estimated affinity constants between 25(OH)D and VDBP and albumin. In the early 2010s, an enzyme-linked immunosorbent assay (ELISA) was developed [21] and became commercially available for the direct measurement of free 25(OH)D. The ELISA is based on monoclonal anti-25(OH)D antibodies and uses a specific incubation buffer that enables the capture of the free fraction of 25(OH)D only [21]. The ELISA was validated and compared with the dialysis technique using 15 samples with a regression line slope of 0.992 and *R*^2^ = 0.737 [21]. This ELISA is currently the only commercially available assay for free 25(OH)D, and it has been used for the direct determination of free 25(OH)D in numerous studies since 2013 as documented in a 2018 review by Tsuprykov et al. [18], where the authors summarized 54 original papers reporting measurements of free 25(OH)D from 1984 to 2017 using either dialysis (4%), calculation (65%), or ELISA (31%) methods. In 2020, Wang et al. [22] reported using a method based on preparation using an ultrafiltration tube, derivatization with PTAD, and finally separation and detection by LC–MS/MS for the assessment of free 25(OH)D. Based on a vitamin D workshop held in 2016 reported by Bikle et al. [15], a clear need for standardization of measurements of VDBP and free 25(OH)D was identified.

To assist in standardization of measurements of 25(OH)D among laboratories and over time, the Office of Dietary Supplements at the National Institutes of Health (NIH-ODS) organized the Vitamin D Standardization Program (VDSP) in 2010 [23, 24], a collaboration among NIH-ODS, the National Institute of Standards and Technology (NIST), the Centers for Disease Control and Prevention (CDC), and national survey laboratories in several countries. A reference measurement system has been established that includes reference measurement procedures (RMPs) at NIST [25, 26], Ghent University [27], and CDC [28]; NIST Standard Reference Materials® (SRMs) [29–31]; the CDC Vitamin D Standardization – Certification Program (VDSCP) [32]; and collaborations with two accuracy-based proficiency testing/external quality assessment (PT/EQA) programs, i.e., the US College of American Pathologists (CAP) accuracy-based vitamin D (ABVD) program [33] and the UK-based Vitamin D External Quality Assessment Scheme (DEQAS) [34, 35].

In 2009, NIST issued the first frozen serum matrix SRM for the determination of vitamin D metabolites including 25(OH)D_2_, 25(OH)D_3_, and 3-epi-25-hydroxyvitamin D_3_, i.e., SRM 972 Vitamin D in Frozen Human Serum with four serum pools with different levels of the metabolites [29]. When the inventory of SRM 972 was exhausted in 2011, an improved SRM 972a Vitamin D Metabolites in Frozen Human Serum was developed again with four different serum pools representing different levels of metabolites [30]. In 2017, SRM 2973 Vitamin D Metabolites in Frozen Human Serum (High Level) was released consisting of one serum pool with a high level of 25(OH)D_3_ (39.4 ng/mL) [31]. In addition to the three metabolites with certified values in SRM 972a, SRM 2973 also had values assigned for 24R,25-dihydroxyvitamin D_3_, and values for this metabolite were also added to SRM 972a at this time. Recently, a unique SRM was produced with serum from female donors of reproductive age who were either not pregnant or pregnant in each of the three trimesters, i.e., SRM 1949 Frozen Prenatal Human Serum [36]. Intended primarily for the determination of thyroid hormones, SRM 1949 also has values assigned for 25(OH)D_2_, 25(OH)D_3_, 3-epi-25-hydroxyvitamin D_3_, and VDBP. These SRMs have found widespread use within the vitamin D testing and research community during the past decade for method development and validation and as control materials for routine testing with over 4500 units distributed to hospital/clinical centers, university, commercial testing, and government laboratories [24].

The components of the reference measurement system for total 25(OH)D as described above follow the recommendations established for harmonization of clinical laboratory measurement procedures [37–40]. However, at present, there is no reference measurement system in place or harmonization efforts underway regarding the measurement of free 25(OH)D. As a first step towards harmonization of measurements of free 25(OH)D, the goal of this study was to determine target values for free 25(OH)D using the ELISA method in existing SRMs used for the determination of vitamin D metabolites. With the availability of target values for free 25(OH)D in these SRMs, laboratories can use these SRMs as controls to assure the quality of their measurements and to validate new methods for determination of free 25(OH)D.

## Experimental

### SRMs evaluated

Three NIST SRMs with values assigned for 25(OH)D_2_, 25(OH)D_3_, and total 25(OH)D were evaluated for the content of free 25(OH)D. SRM 972a Vitamin D Metabolites in Frozen Human Serum (3 units, 1 vial per level) [30] and 2973 Vitamin D Metabolites in Frozen Human Serum (High Level) (2 units, 2 vials per level) [31] were provided by NIST and shipped on dry ice to the two participating laboratories. SRM 1949 Frozen Human Prenatal Serum (2 units, 2 vials per level) [36] was purchased from NIST and shipped on dry ice to the two participating laboratories. The SRMs were then stored at − 70 °C at each laboratory until analyzed.

### Single-donor samples evaluated and selection of laboratories for the evaluation of SRMs

Prior to this study, an interlaboratory comparison study of the performance of the free 25(OH)D ELISA was conducted among nine laboratories using the ELISA to determine free 25(OH)D. Forty single-donor samples were analyzed from a 50 single-donor sample set used in an earlier interlaboratory comparison exercise for the determination of total 25(OH)D described in Wise et al. [6] with values for total 25(OH)D ranging from 6.5 to 61.1 ng/mL. The results of the analysis of these samples for the determination of 25(OH)D_2_ and 25(OH)D_3_ using the NIST reference measurement procedures [25] were reported previously [6]. These 40 samples were analyzed for the determination of free 25(OH)D at the Nutritional Biomarker Laboratory, Medical Research Council (MRC) Epidemiology Unit at the University of Cambridge (Cambridge, UK), using the approach described below. The results from the interlaboratory comparison informed the selection of the laboratories for the evaluation of SRMs in the study reported here.

### Directly measured free 25(OH)D procedures

Two laboratories, Future Diagnostics (Wijchen, The Netherlands) and Nutritional Biomarker Laboratory (NBL), MRC Epidemiology Unit at the University of Cambridge (Cambridge, UK), analyzed the SRM samples using the DIAsource Free 25OH Vitamin D ELISA assay. The SRM samples were run on each of 3 days with two runs per day and two replicates per run (i.e., 3 days × 2 runs/day × 2 replicates per run = 12 determinations per each level of each SRM). Only one SRM vial was used per day to avoid any potential freeze/thaw stability concerns.

The free 25(OH)D ELISA is based on a two-step immunoassay performed in a microtiter plate. During the first step, free 25(OH)D_2_ and 25(OH)D_3_ bind with the anti-vitamin D antibody coated on the bottom of the microtiter plate wells. After washing, a fixed amount of biotinylated 25(OH)D is added to each well to react with the unoccupied antibody binding sites. After washing to remove unbound biotinylated 25(OH)D, streptavidin–peroxidase conjugate is added, and the bound enzyme is quantified colorimetrically [21].

Both laboratories followed the manufacturer’s instructions [21] for measurement of free 25(OH)D using the DIAsource assay. DIAsource Free 25OH Vitamin D ELISA assay short instructions are as follows (KAPF1991, DiaSource ImmunoAssays S.A., Louvain-la Neuve, BE). Add 90 μL sample diluent to the wells. Transfer 10 μL calibrator, control, or sample in duplicate into the appropriate well of the microtiterplate. Incubate the plate for 90 min at 37 °C while shaking at 650 rpm. Wash the plate 3 times with wash buffer. Add 100 μL Biot-VitD reagent to the wells. Incubate the plate for 30 min at 37 °C while shaking at 650 rpm. Wash the plate 3 times with wash buffer. Add 100 μL streptavidin-HRP reagent to the wells. Incubate the plate for 20 min at 37 °C while shaking at 650 rpm. Wash the plate 3 times with wash buffer. Add 100 μL substrate reagent to the wells. Incubate the plate for 15 min at room temperature while stationary and protected from light. Add 100 μL stop reagent to each well. Read the plate at 450 nm within 5 min. The information regarding instruments, calibrators, and instrument controls used by both laboratories are provided in Tables S1, S2, and S3 (Supplementary Information), respectively, and are described briefly below.

#### Nutritional Biomarker Laboratory (NBL), University of Cambridge

The free 25(OH)D ELISA was performed according to the manufacturer’s instructions by a single analyst. Incubation and shaking were performed with a BMG THERMOstar (BMG Labtech Ltd., UK) and washing with a Thermo Wellwash (Thermo Fisher Scientific, UK). Results were read using a Thermo Multiskan (Thermo Fisher Scientific, UK).

#### Future Diagnostics

The free 25(OH)D ELISA was performed according to the manufacturer’s instruction by three different analysts. A Thermo iEMS shaker incubator (Thermo Scientific, USA) was used for the incubation, washing was done using a Biotek elx50, and absorption values were measured using a Biotek elx800 plate reader (Biotek, USA).

### Statistical methods

All data analyses were conducted using CBStat 5.1 (Copyright by Kristian Linnet, Ordup Have 15, DK-2920 Charlottenlund, Denmark). Paired *t* test was used to test for significant differences between Future Diagnostics and NBL Cambridge determinations for day, run, and duplicate. Mean paired difference over day, run, and duplicate was calculated as the NBL Cambridge determination minus the Future Diagnostics determination. Ordinary least squares regression analysis was used to compare mean values for each SRM level (*n* = 9).

## Results and discussion

### Interlaboratory comparison and selection of laboratories for the evaluation of SRMs

For the interlaboratory comparison study conducted among nine laboratories using the ELISA to determine free 25(OH)D in a set of 40 single-donor samples [41], overall %CVs for the individual laboratories ranged from 2.8 to 22% with only the assay manufacturer laboratory (Future Diagnostics) and NBL Cambridge achieving %CVs below 4%. The results obtained by Future Diagnostics were considered to be the benchmark, and bias compared to the Future Diagnostics results ranged from 1.3 to 29% with only two laboratories achieving a bias below 5% [41]. The results of this interlaboratory study indicated a need for improvement among laboratories in using this ELISA for the determination of free 25(OH)D. Based on the results of the interlaboratory comparison study, NBL Cambridge and Future Diagnostics were selected to analyze the SRM suite to assign target values for free 25(OH)D using the ELISA.

**Fig. 1:**
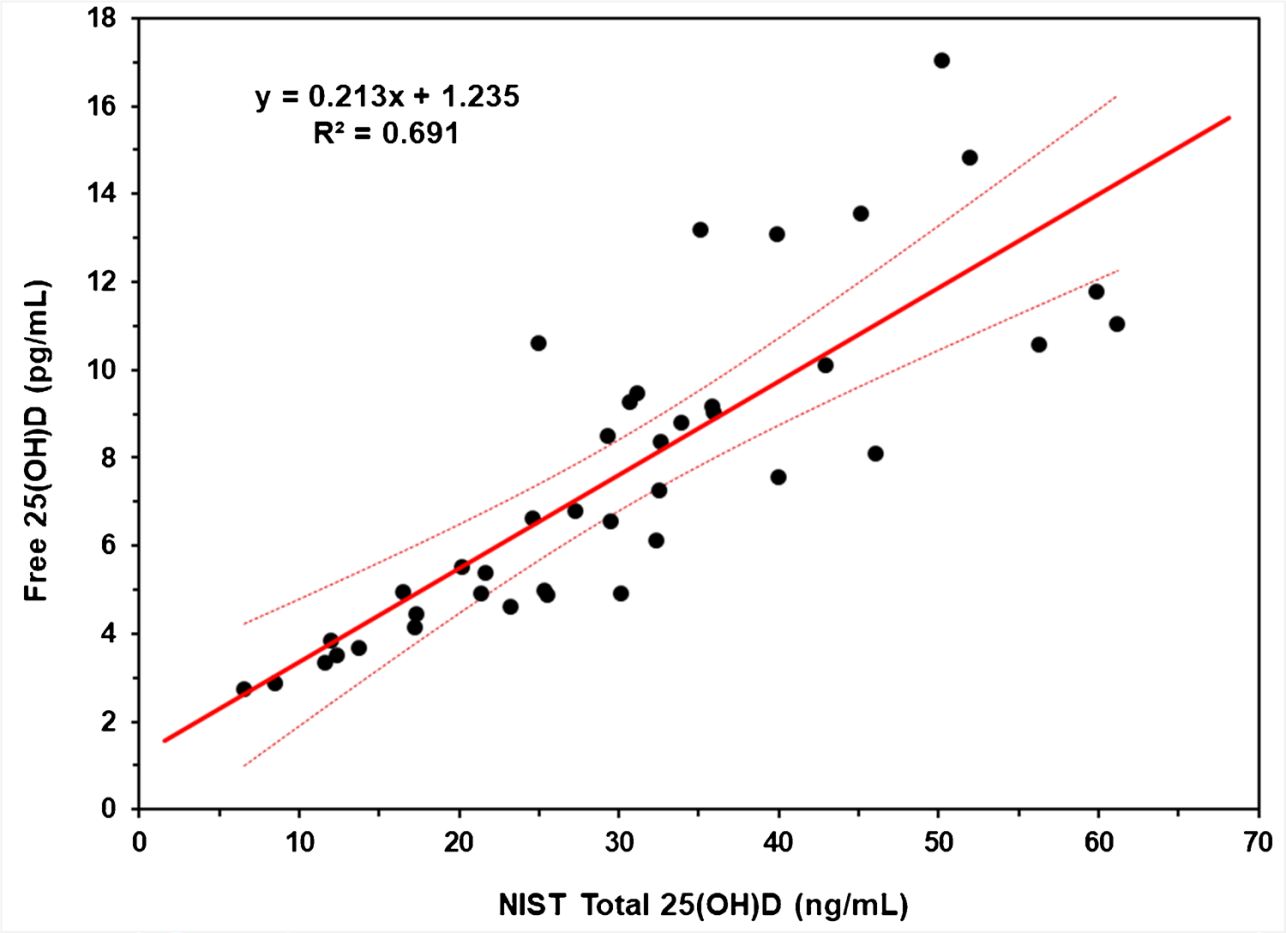
Ordinary least squares linear regression for free 25(OH)D and total 25(OH)D in 40 single-donor patient samples. Black circles are the single-donor samples, and the solid red line is the regression line. The red dashed line is the 95% confidence interval for the regression line. Free 25(OH)D measurements performed at NBL Cambridge

The NBL Cambridge results for the determination of free 25(OH)D in the 40 single-donor samples are summarized in Table S4. Similar results from the analysis of the same samples by Future Diagnostics are provided in Table S5. These results illustrate the repeatability achievable when using the ELISA. A plot of the free 25(OH)D (NBL Cambridge) concentration versus the NIST Total 25(OH)D in the 40 single-donor samples is shown in Fig. 1, and a similar plot using the Future Diagnostics results for free 25(OH)D is provided in Figure S1 (Supplementary Information).

### Comparison and combination of results from two laboratories

Three NIST SRMs with nine different levels were available with values assigned for 25(OH)D_2_, 25(OH)D_3_, total 25(OH)D, and VDBP as summarized in Table 1. Total 25(OH)D concentrations for the nine materials range from 18.9 to 40.1 ng/mL, with 25(OH)D_3_ ranging from 18.1 to 39.4 ng/mL. For the four levels of SRM 1949, values are assigned for VDBP, which increases in concentration from 211.5 μg/mL in the non-pregnant serum pool to 383.4 μg/mL in the third trimester serum pool.

**Table 1:** National Institute of Standards and Technology (NIST) Standard Reference Materials (SRM®) with vitamin D metabolite assigned values and uncertainty used in this study

SRM samples	Serum vitamin D metabolite^a^ (ng/mL)						Vitamin D binding protein (μg/mL)	
	25(OH)D_2_		25(OH)D_3_		Total 25(OH)D			
	Mean	Uncertainty	Mean	Uncertainty	Mean	Uncertainty	Mean	Uncertainty
SRM 972a level 1	0.54	0.06	**28.8**	**1.1**	29.3	1.1	NR^b^	NR
SRM 972a level 2	**0.81**	**0.06**	**18.1**	**0.4**	**18.9**	**0.4**	NR	NR
SRM 972a level 3	**13.2**	**0.3**	**19.8**	**0.4**	**33.0**	**0.5**	NR	NR
SRM 972a level 4	0.55	0.10	**29.4**	**0.9**	30.0	0.9	NR	NR
SRM 2973	0.65	0.02	**39.4**	**0.8**	40.1	0.8	NR	NR
SRM 1949 NP^c^	0.278	NR	24.98	0.28	25.3^d^	NR	211.5	2.8
SRM 1949 1T^c^	1.20	0.05	26.01	0.22	27.2^d^	NR	286.7	3.8
SRM 1949 2T^c^	0.514	0.037	30.00	0.50	30.5^d^	NR	349.7	4.3
SRM 1949 3T^c^	0.897	0.057	29.43	0.41	30.3^d^	NR	383.4	5.1

^a^Certified values in bold type; reference values in normal type. Uncertainties for certified and reference values are not equivalent; see SRM Certificate of Analysis (COA) for complete description of the uncertainties of certified and reference values. To convert ng/mL to nmol/L, multiply 25(OH)D_2_ concentration by 2.423 and 25(OH)D_3_ concentration by 2.496

^b^*NR*, not reported

^c^*NP*, non-pregnant; *1T*, 1st trimester of pregnancy; *2T*, 2nd trimester of pregnancy; and *3T*, 3rd trimester of pregnancy

^d^Values calculated for this study and not assigned by NIST on the SRM COA

**Fig. 2:**
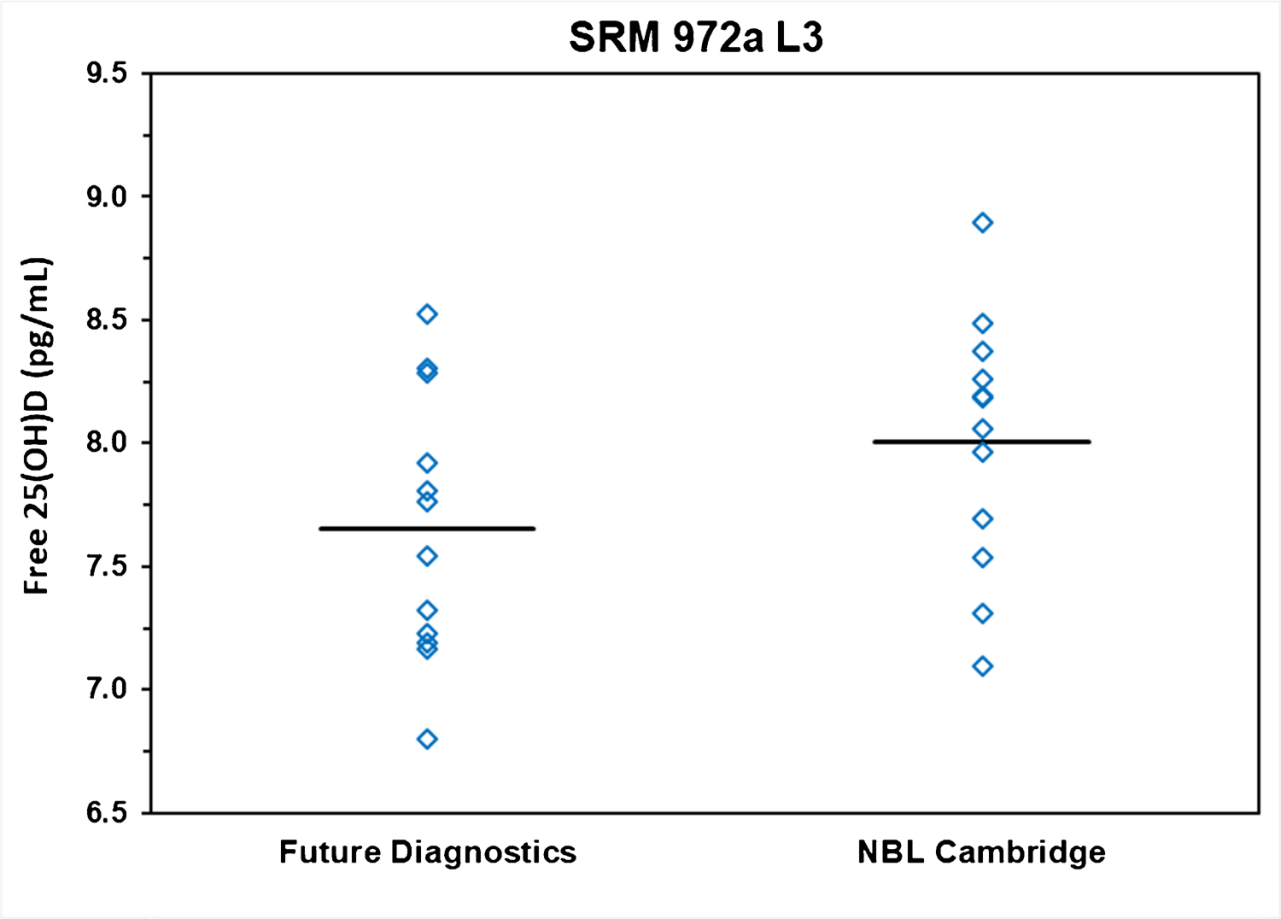
Distribution of free 25(OH)D (pg/mL) measurements by laboratory for NIST SRM 972a — Vitamin D Metabolites in Frozen Human Serum (level 3). Blue diamonds are the individual measurements. Black bar is the mean value of the 12 measurements

**Fig. 3:**
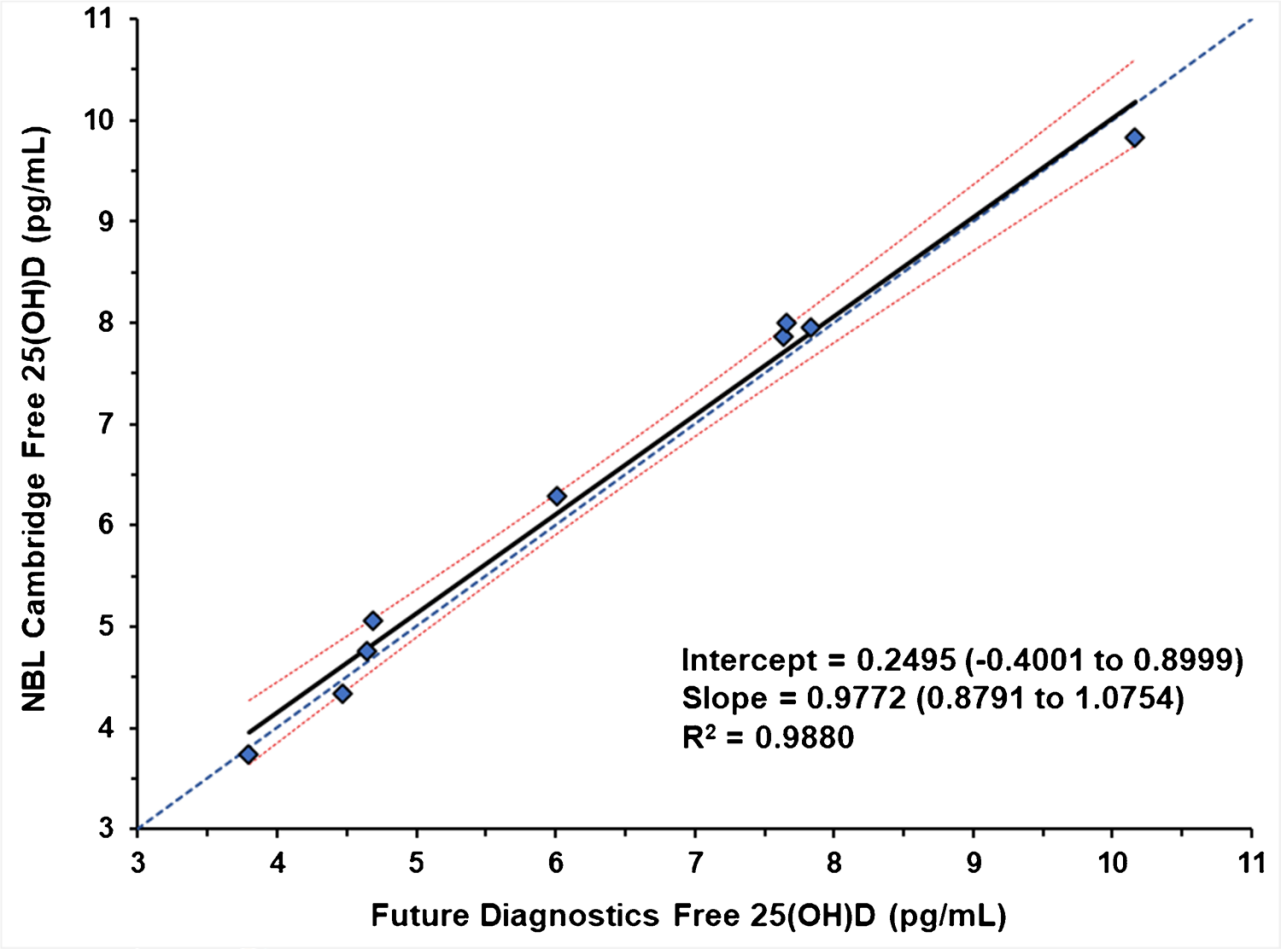
Ordinary least squares regression of the means for directly measured free 25(OH)D concentrations by laboratory. The black solid line is the regression line; the dashed blue line is the identity line (*y* = x); and the red dashed lines are the 95% confidence interval for the regression line

All nine levels of the SRMs were analyzed to determine the content of free 25(OH)D using the ELISA in two laboratories. The individual results (*n* = 12 for each SRM level) from the two laboratories for the determination of free 25(OH)D are provided in Table 2. The paired mean differences between NBL Cambridge and Future Diagnostics measurements were small as shown in Table 3. The results from the two laboratories were combined, and the means and standard deviations are summarized in Table 4. In six of the nine NIST SRM levels, the NBL Cambridge measurements were slightly higher than the Future Diagnostics Solutions measurements. None of the paired differences were statistically significant (*p* < 0.05) and the 95% confidence limits for the mean difference in every case included zero. A distribution plot of the results from both laboratories for SRM 972 level 3 is shown in Fig. 2. Similar distribution plots for the remaining SRM samples are provided in Figures S2 to S9 in Supplementary Information. A plot of the least squares regression of the directly measured free 25(OH)D means from the two laboratories is shown in Fig. 3. The regression line with *R*^2^ = 0.99 and slope of 0.977 with 95% confidence interval including zero intercept and slope of 1.00 indicate the near perfect correspondence between the two laboratories for the direct measurement of free 25(OH)D. The standardized residual plot for the regression model shown in Figure S10 (Supplementary Information) does not indicate any violation of the regression model assumptions.

**Table 2:** Individual measurement results from both laboratories for the three SRMs

				SRM 972a				SRM 2973	SRM 1949			
				Level 1	Level 2	Level 3	Level 4		NP^a^	1st	2nd	3rd
Lab^b^	Day	Run	Rep^c^	Free 25(OH)D_3_ (pg/mL)^d^								
1	1	1	1	7.750	5.341	8.261	8.034	9.742	6.222	4.331	3.471	3.483
1	1	1	2	8.148	5.164	7.313	7.528	9.815	6.133	4.677	4.566	3.681
1	1	2	1	6.966	4.991	8.191	8.401	10.263	6.171	3.771	3.986	3.288
1	1	2	2	7.917	4.210	7.691	8.442	10.479	6.564	5.004	5.242	4.288
1	2	1	1	7.579	5.898	8.893	8.326	10.372	6.230	4.952	3.465	3.972
1	2	1	2	7.841	5.186	7.538	7.700	9.678	6.437	5.087	4.668	3.982
1	2	2	1	7.354	5.694	8.373	8.394	9.501	6.680	4.755	4.406	3.450
1	2	2	2	7.807	4.975	7.967	8.411	9.840	6.368	4.935	4.840	3.847
1	3	1	1	8.143	5.208	8.187	8.038	9.535	6.001	4.987	4.104	4.013
1	3	1	2	7.655	4.185	7.096	7.096	9.264	5.955	4.875	4.783	3.392
1	3	2	1	7.783	5.083	8.057	7.765	9.866	6.145	4.840	4.051	3.577
1	3	2	2	9.385	4.847	8.485	7.383	9.631	6.532	4.948	4.440	3.846
2	1	1	1	7.421	4.817	7.923	7.470	10.022	6.161	4.610	4.116	3.712
2	1	1	2	6.800	4.408	7.193	8.040	9.683	5.746	4.458	4.375	3.826
2	1	2	1	8.704	5.788	8.302	8.665	10.995	6.667	5.216	5.360	4.359
2	1	2	2	8.472	5.223	8.283	8.892	11.704	6.740	5.795	5.066	4.271
2	2	1	1	8.113	4.719	7.230	7.680	9.231	5.985	4.441	3.891	2.750
2	2	1	2	7.168	3.960	7.168	7.116	9.886	5.397	4.108	4.101	3.553
2	2	2	1	7.177	4.719	7.324	7.497	9.458	5.534	4.360	4.145	3.600
2	2	2	2	7.063	4.363	6.799	7.344	9.609	5.504	4.350	4.472	3.955
2	3	1	1	8.181	5.231	8.525	8.716	10.904	6.371	4.685	4.497	3.732
2	3	1	2	7.348	3.435	7.765	7.668	10.215	6.392	4.878	4.497	3.711
2	3	2	1	7.524	5.014	7.807	7.708	10.120	5.676	4.630	4.328	3.785
2	3	2	2	7.643	4.616	7.545	7.189	10.134	5.950	4.161	4.779	4.293

^a^Non-pregnant, 1st trimester, 2nd trimester, and 3rd trimester

^b^1, Nutritional Biomarker Laboratory (NBL), Cambridge; and 2, Future Diagnostics Laboratory

^c^*Rep*, replicate

^d^To convert pg/mL to pmol/L, multiply by 2.496

**Table 3:** Comparison of paired mean directly measured free 25(OH)D

SRMs®	University of Cambridge		Future Diagnostics Solutions		Combined		Paired *t* test	
	Concentration (pg/mL)							95% CL for
	Mean	SD	Mean	SD	Mean	SD	Mean difference^a^	Mean difference
SRM 972a level 1	7.86	0.58	7.63	0.60	7.75	0.59	0.23	− 0.35 to 0.81
SRM 972a level 2	5.06	0.50	4.69	0.62	4.88	0.58	0.37	− 0.08 to 0.83
SRM 972a level 3	8.00	0.50	7.66	0.54	7.83	0.54	0.34	− 0.12 to 0.81
SRM 972a level 4	7.96	0.46	7.83	0.61	7.90	0.53	0.13	− 0.26 to 0.52
SRM 2973	9.83	0.37	10.16	0.72	10.00	0.58	− 0.33	− 0.77 to 0.11
SRM 1949 NP^b^	6.29	0.23	6.01	0.46	6.15	0.38	0.28	− 0.09 to 0.64
SRM 1949 1T^b^	4.76	0.37	4.64	0.47	4.70	0.42	0.12	− 0.31 to 0.56
SRM 1949 2T^b^	4.34	0.54	4.47	0.42	4.40	0.48	− 0.13	− 0.48 to 0.21
SRM 1949 3T^b^	3.74	0.30	3.80	0.43	3.76	0.36	− 0.06	− 0.41 to 0.29

^a^Difference is NBL Cambridge (pg/mL) – Future Diagnostics Solutions (pg/mL)

^b^NP, non-pregnant; 1T, 1st trimester of pregnancy; 2T, 2nd trimester of pregnancy; and 3T, 3rd trimester of pregnancy

^c^To convert pg/mL to pmol/L multiply by 2.496, except for SRM 972a level 3 which has a high concentration of serum 25(OH)D_2_

**Table 4:** Summary of percent of free 25(OH)D_3_ in the SRMs

	Free 25(OH)D_3_ (pg/mL)		Total 25(OH)D (ng/mL)		Percent free 25(OH)D	
	Mean	SD	Mean	Uncertainty^a^	Percent^b^	SD^b^
SRM 972a level 1	7.75	0.59	29.3	1.1	0.0264	0.0020
SRM 972a level 2	4.88	0.58	18.9	0.4	0.0258	0.0031
SRM 972a level 3	7.83	0.54	33.0	0.5	0.0237	0.0016
SRM 972a level 4	7.90	0.53	30.0	0.9	0.0264	0.0018
SRM 2973	10.00	0.58	40.1	0.8	0.0250	0.0015
SRM 1949 NP^c^	6.15	0.38	25.3^d^	NR^e^	0.0246	0.0015
SRM 1949 1T^c^	4.70	0.42	27.2^d^	NR^e^	0.0181	0.0016
SRM 1949 2T^c^	4.40	0.48	30.5^d^	NR^e^	0.0144	0.0016
SRM 1949 3T^c^	3.76	0.36	30.3^d^	NR^e^	0.0124	0.0012

^a^Uncertainty from the NIST SRM COA

^b^Mean of free 25(OH)D from both laboratories/total 25(OH)D NIST × 100; *SD*, standard deviation of combined measurements from both laboratories

^c^NP, non-pregnant; 1T, 1st trimester of pregnancy; 2T, 2nd trimester of pregnancy; and 3T, 3rd trimester of pregnancy

^d^Values calculated [sum of 25(OH)D_2_ and 25(OH)D_3_] for this study and not assigned by NIST on the SRM COA

^e^*NR*, not reported on COA

**Fig. 4:**
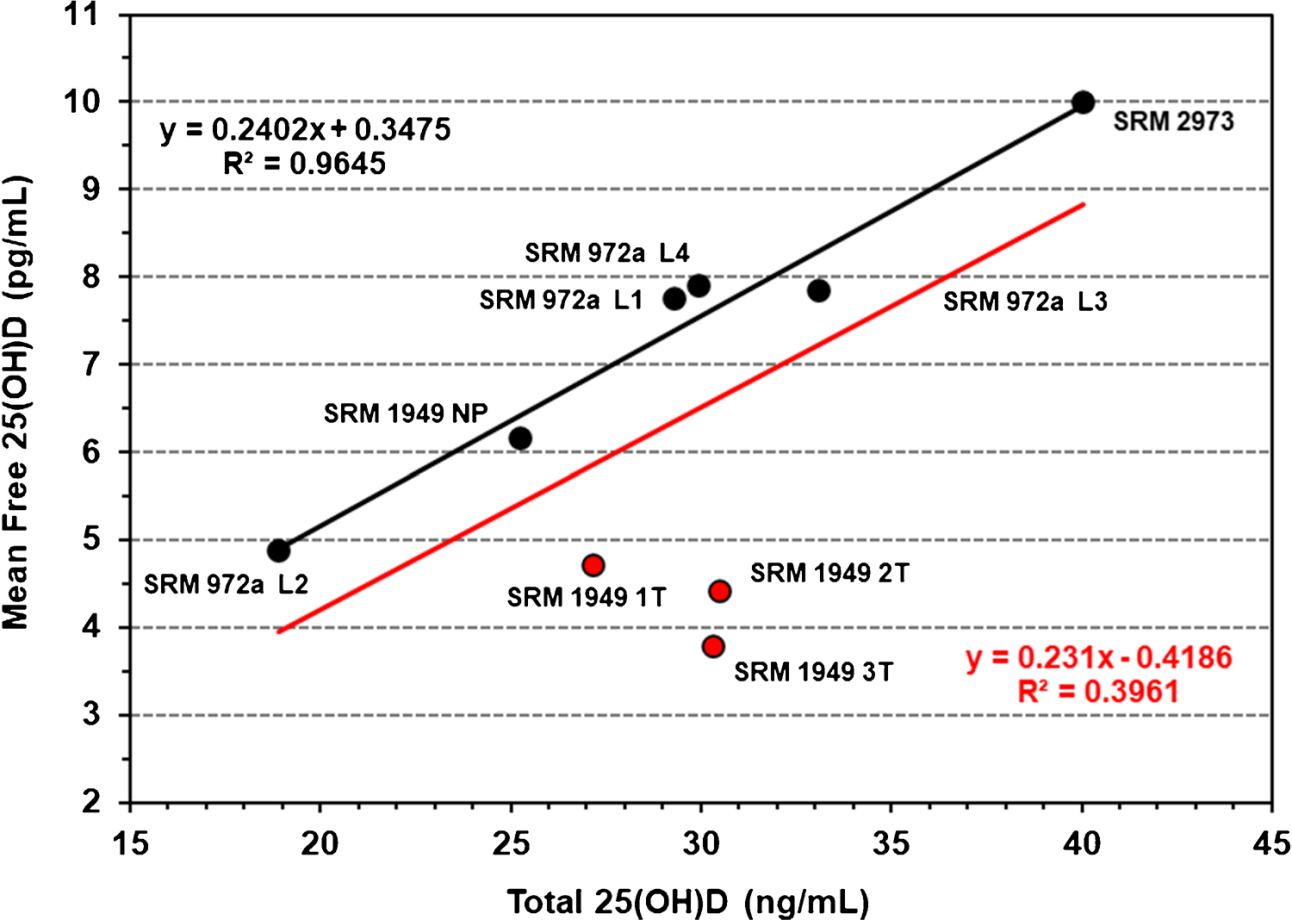
Least squares linear regression for free 25(OH)D and total 25(OH)D for nine SRM levels. The black dots and black regression line are SRM 972a, SRM 2973, and SRM 1949 (non-pregnant level); the red dots are for SRM 1949 levels representing the 1st, 2nd, and 3rd trimester serum pools, and the red regression line is for all samples. Regression equation and *R*^2^ value in black are for SRM 972a, SRM 2973, and SRM 1949 (non-pregnant level); red regression equation and *R*^2^ value are for all samples

**Fig. 5:**
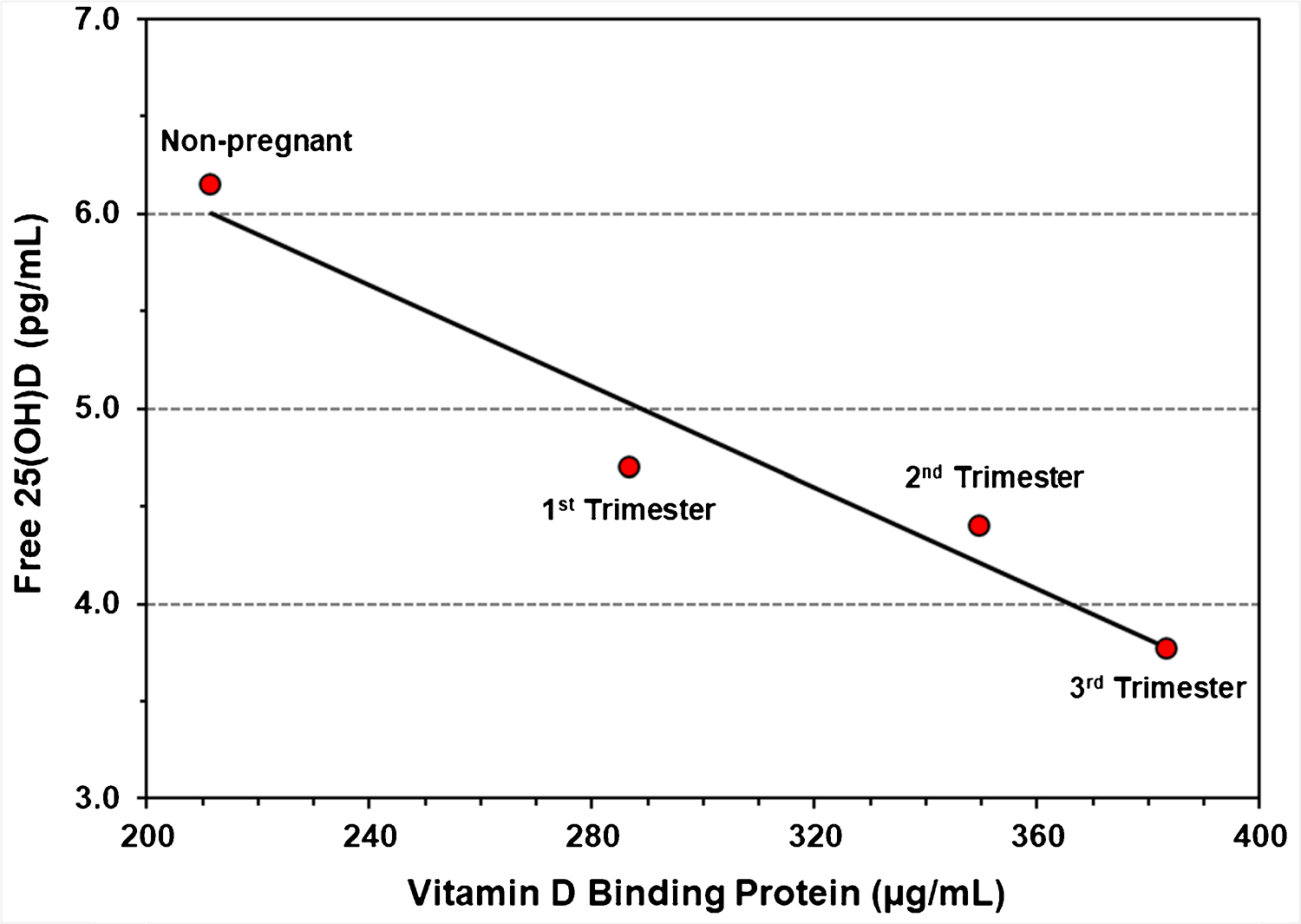
Least squares linear regression for free 25(OH)D versus vitamin D binding protein for different SRM 1949 levels

The correlation of directly measured free 25(OH)D with the total 25(OH)D content for the nine SRM levels is shown in Fig. 4. The relationship between free 25(OH)D and total 25(OH)D in the three levels in SRM 1949 representing the three pregnancy trimesters is obviously different from the other SRM serum pools as indicated in Fig. 4 (red dots), and the percent free 25(OH)D was lower in pregnancy compared to non-pregnancy samples. This observation may be explained by the higher circulating level of VDBP observed in pregnant compared to non-pregnant women [42–45] in SRM 1949 where VDBP concentration is associated with directly measured free 25(OH)D concentration between non-pregnancy and pregnancy and in the increased VDBP concentration across gestation (Fig. 5) as reported in longitudinal studies during pregnancy [42, 45]. However, Bikle and Swartz [17] suggest that free 25(OH)D may be the same or only slightly lower during pregnancy.

### Use of SRMs for harmonization of measurements

The assignment of target values for free 25(OH)D using the ELISA assay in existing SRMs is an initial step towards harmonization and eventual standardization of measurements of free 25(OH)D. These SRMs are available to any researcher for use as control samples or for assigning target values to in-house control materials. The use of these SRMs for reporting free 25(OH)D concentrations in various studies will allow for comparison of results among different laboratories, different studies, and over time. We recommend that users of the ELISA for free 25(OH)D analyze one or more of the SRMs and compare their results with the target values reported in this paper to determine laboratory/assay performance. If the results are biased, users should evaluate whether the assay protocol has been appropriately followed. Results among different laboratories, and different studies can be harmonized using approaches as described previously for 25(OH)D assays [46, 47].

The availability of additional information characterizing these existing SRMs for vitamin D metabolites enhances the value of these materials [48]. In particular, the addition of free 25(OH)D results to SRM 1949 provides valuable information on the effect of changes in VDBP concentration on free 25(OH)D in pregnancy. This information may also be relevant to other pathological conditions associated with changes in VDBP concentration [49] and their effect on vitamin D metabolism.

## Supplementary Information

Below is the link to the electronic supplementary material.

Supplementary file1 (DOCX 297 KB)
