# Supplementary material for: Towards harmonization of directly measured free 25-hydroxyvitamin D using an enzyme-linked immunosorbent assay
Source: Anal Bioanal Chem. 2022 Sep 16;414(27):7793–803. doi: 10.1007/s00216-022-04313-y (PMC9568476; doi:10.1007/s00216-022-04313-y)

**Supplementary Information**

**Analytical and Bioanalytical Chemistry**

**Towards Harmonization of Directly-Measured Free 25-Hydroxyvitamin D Using an Enzyme-linked Immunosorbent Assay**

Christopher T. Sempos^1,2^, Ernst Lindhout^3^, Nicolas Heureux^4,5^, Michel Hars^4^, Damon A. Parkington^6^, Emily Dennison^7^, Ramón Durazo-Arvizu^8^, Kerry S. Jones^6*^ and Stephen A. Wise^1^

^1^ Office of Dietary Supplements, National Institutes of Health, Bethesda, MD 20892, USA

^2^ Vitamin D Standardization Program, LLC, Havre de Grace, MD 21078, USA

^3^ Future Diagnostics, Nieuweweg 279, 6603 BN Wijchen, The Netherlands

^4^ DIAsource Immunoassays, B-1348 Louvain-La-Neuve, Belgium

^5^ BioSweet srl, B-5030 Lonzee, Belgium

^6^ Nutritional Biomarker Laboratory, MRC Epidemiology Unit, University of Cambridge, Cambridge CB2 0AH, United Kingdom

^7^ Immuno-Biological Laboratories Inc (IBL-America), Minneapolis, MN 55432, USA

^8^ The Saban Research Institute, Children's Hospital Los Angeles, Los Angeles, CA 90027, USA

*Corresponding author: kerry.jones@mrc-epid.cam.ac.uk

Table S1. Instrumentation Used by Participating Laboratories in this Study

| **Instrumentation/Assay** | **Future Diagnostics Solutions** | **Nutritional Biomarker Laboratory (NBL), University of Cambridge** |
| --- | --- | --- |
| Free 25(OH)D method | Free 25OH Vitamin D ELISA | Free 25OH Vitamin D ELISA |
|  |  |  |
| 37 °C incubator | Shaker-incubator from Thermo | BMG THERMOstar |
| Reader | Biotek elx800 | Thermo Multiskan |
| Washer | Biotek elx50 | Thermo Wellwash |
| Shaker | Shaker-incubator from Thermo | BMG THERMOstar |
| Volume of serum used per analysis | 10 µL | 10 µL |
| Analysis Time per sample | 0.52 s per well | 0.52 s per well |

Table S2. Instrument Controls Used by Both Laboratories in this Study

| **Manufacturer** | **Future Diagnostics** | **NBL Cambridge** | **Control ID** | **Acceptable concentration range** | | **Future Diagnostics** | **NBl Cambridge** |
| --- | --- | --- | --- | --- | --- | --- | --- |
|  | **Lot No.** | |  | **Lower (pg/mL)** | **Upper (pg/mL)** | **Free 25(OH)D (pg/mL)** | |
| Day 1, Run 1 | M68132 | M68132 |  |  |  |  |  |
|  | M68132 | M67415 | CTRL-1 | 3.2 | 6.0 | 4.7 | 5.4 |
|  | M68132 | M67340 | CTRL-2 | 11.4 | 21.4 | 16.4 | 14.9 |
| Day 1, Run 2 | M68132 | M68132 |  |  |  |  |  |
|  | M68132 | M67415 | CTRL-1 | 3.2 | 6.0 | 5.0 | 4.7 |
|  | M68132 | M67340 | CTRL-2 | 11.4 | 21.2 | 17.6 | 15.7 |
| Day 2, Run 1 | M68132 | M68132 |  |  |  |  |  |
|  | M68132 | M67415 | CTRL-1 | 3.2 | 6.0 | 4.1 | 5.6 |
|  | M68132 | M67340 | CTRL-2 | 11.4 | 21.2 | 17.4 | 15.2 |
| Day 2, Run 2 | M68132 | M68132 |  |  |  |  |  |
|  | M68132 | M67415 | CTRL-1 | 3.2 | 6.0 | 4.1 | 5.2 |
|  | M68132 | M67340 | CTRL-2 | 11.4 | 21.2 | 16.1 | 16.0 |
| Day 3, Run 1 | M68132 | M68132 |  |  |  |  |  |
|  | M68132 | M67415 | CTRL-1 | 3.2 | 6.0 | 4.6 | 5.4 |
|  | M68132 | M67340 | CTRL-2 | 11.4 | 21.2 | 18.3 | 15.2 |
| Day 3, Run 2 | M68132 | M68132 |  |  |  |  |  |
|  | M68132 | M67415 | CTRL-1 | 3.2 | 6.0 | 4.8 | 5.3 |
|  | M68132 | M67340 | CTRL-2 | 11.4 | 21.2 | 16.1 | 15.8 |

Table S3. Calibrator Information for Free 25(OH)D Assay Used by Both Laboratories in this Study^a^

|  | **Analyte** | **Manufacturer** | **Lot No.** | **Designation** | **Concentration (pg/mL)** |
| --- | --- | --- | --- | --- | --- |
| Calibrator Level 1 | Free 25-hydroxyvitamin D | Future Diagnostics | M67333 | CAL A | 0.9 |
| Calibrator Level 2 | Free 25-hydroxyvitamin D | Future Diagnostics | M67334 | CAL B | 3.1 |
| Calibrator Level 3 | Free 25-hydroxyvitamin D | Future Diagnostics | M67335 | CAL C | 6.5 |
| Calibrator Level 4 | Free 25-hydroxyvitamin D | Future Diagnostics | M67336 | CAL D | 11.6 |
| Calibrator Level 5 | Free 25-hydroxyvitamin D | Future Diagnostics | M67337 | CAL E | 23.2 |
| Calibrator Level 6 | Free 25-hydroxyvitamin D | Future Diagnostics | M67338 | CAL F | 40.3 |

^a^ Same calibrators were used for runs 1 and 2 on days 1, 2, and 3.

Table S4. Measurements of Free 25(OH)D using the ELISA assay in 40 patient samples at NBL Cambridge compared with 25(OH)D concentration determined using a Reference Measurement Procedure

| **Sample No.** | **Free 25(OH)D (pg/mL)** | | | | | | | | | | **Total**  **25(OH)D**  **(ng/mL)^a^** | **Percent**  **Free 25(OH)D** |
| --- | --- | --- | --- | --- | --- | --- | --- | --- | --- | --- | --- | --- |
|  | **Day 1** | | | **Day 2** | | **Day 3** | |  | | |  |  |
|  | **Rep 1** | **Rep 2** | **Rep 1** | | **Rep 2** | **Rep 1** | **Rep 2** | **Mean** | **SD** | **CV** |  |  |
| 1 | 12.1 | 11.0 | 11.0 | | 10.4 | 11.6 | 10.3 | 11.1 | 0.7 | 6.3 | 61.1 | 0.0182 |
| 2 | 12.7 | 11.6 | 11.7 | | 11.3 | 12.3 | 11.1 | 11.8 | 0.6 | 5.2 | 59.8 | 0.0197 |
| 3 | 11.3 | 10.3 | 10.8 | | 9.90 | 11.0 | 10.3 | 10.6 | 0.5 | 4.9 | 56.2 | 0.0189 |
| 4 | 13.8 | 12.7 | 13.8 | | 13.1 | 14.2 | 13.8 | 13.6 | 0.6 | 4.1 | 45.1 | 0.0302 |
| 5 | 10.3 | 9.51 | 10.4 | | 10.1 | 10.4 | 10.0 | 10.1 | 0.3 | 3.4 | 42.9 | 0.0235 |
| 6 | 14.1 | 12.1 | 13.7 | | 13.0 | 13.5 | 12.9 | 13.2 | 0.7 | 5.3 | 35.1 | 0.0376 |
| 7 | 9.16 | 7.81 | 8.88 | | 8.09 | 8.74 | 8.33 | 8.5 | 0.5 | 6.0 | 29.2 | 0.0291 |
| 8 | 10.7 | 8.83 | 9.62 | | 8.58 | 10.0 | 9.23 | 9.5 | 0.8 | 8.3 | 31.1 | 0.0305 |
| 9 | 2.62 | 2.90 | 2.63 | | 2.56 | 2.93 | 2.90 | 2.8 | 0.2 | 6.2 | 6.5 | 0.0431 |
| 10 | 9.18 | 8.95 | 9.05 | | 9.08 | 9.54 | 9.28 | 9.2 | 0.2 | 2.3 | 35.8 | 0.0257 |
| 11 | 9.11 | 8.53 | 8.74 | | 9.41 | 8.99 | 9.52 | 9.1 | 0.4 | 4.2 | 35.9 | 0.0253 |
| 12 | 8.54 | 8.32 | 8.37 | | 8.28 | 8.48 | 8.31 | 8.4 | 0.1 | 1.2 | 32.5 | 0.0258 |
| 13 | 9.37 | 8.81 | 9.44 | | 9.29 | 9.06 | 9.79 | 9.3 | 0.3 | 3.6 | 30.6 | 0.0304 |
| 14 | 5.44 | 5.11 | 5.49 | | 5.12 | 5.57 | 5.67 | 5.4 | 0.2 | 4.3 | 21.6 | 0.0250 |
| 15 | 6.94 | 6.33 | 6.25 | | 6.37 | 6.95 | 6.55 | 6.6 | 0.3 | 4.7 | 29.4 | 0.0224 |
| 16 | 10.9 | 10.4 | 10.5 | | 10.2 | 11.1 | 10.7 | 10.6 | 0.3 | 3.1 | 24.9 | 0.0426 |
| 17 | 3.54 | 3.64 | 3.25 | | 3.82 | 4.11 | 3.81 | 3.7 | 0.3 | 7.9 | 13.6 | 0.0272 |
| 18 | 4.64 | 4.48 | 4.11 | | 4.25 | 4.66 | 4.65 | 4.5 | 0.2 | 5.3 | 17.3 | 0.0260 |
| 19 | 16.3 | 16.8 | 16.6 | | 16.5 | 17.9 | 18.2 | 17.1 | 0.8 | 4.7 | 50.2 | 0.0341 |
| 20 | 4.96 | 4.59 | 5.18 | | 4.71 | 5.02 | 4.89 | 4.9 | 0.2 | 4.4 | 25.5 | 0.0192 |
| 21 | 7.24 | 7.28 | 7.37 | | 6.97 | 7.32 | 7.48 | 7.3 | 0.2 | 2.4 | 32.5 | 0.0225 |
| 22 | 3.59 | 3.50 | 3.36 | | 3.35 | 3.70 | 3.75 | 3.5 | 0.2 | 4.8 | 12.3 | 0.0285 |
| 23 | 4.31 | 3.90 | 4.15 | | 4.13 | 4.33 | 4.18 | 4.2 | 0.2 | 3.7 | 17.2 | 0.0244 |
| 24 | 3.58 | 3.18 | 3.44 | | 2.95 | 3.44 | 3.49 | 3.3 | 0.2 | 7.0 | 11.6 | 0.0284 |
| 25 | 4.75 | 4.98 | 4.68 | | 4.85 | 5.26 | 5.12 | 4.9 | 0.2 | 4.5 | 30.1 | 0.0163 |
| 26 | 4.02 | 3.95 | 3.54 | | 3.76 | 4.06 | 3.91 | 3.9 | 0.2 | 5.0 | 12.0 | 0.0325 |
| 27 | 8.34 | 8.49 | 8.68 | | 8.90 | 9.39 | 9.04 | 8.8 | 0.4 | 4.4 | 33.8 | 0.0260 |
| 28 | 5.03 | 4.65 | 4.60 | | 5.05 | 5.35 | 4.89 | 4.9 | 0.3 | 5.7 | 21.3 | 0.0230 |
| 29 | 5.37 | 5.52 | 5.52 | | 5.66 | 5.65 | 5.57 | 5.5 | 0.1 | 1.9 | 20.2 | 0.0272 |
| 30 | 6.65 | 6.47 | 6.86 | | 6.42 | 6.60 | 6.86 | 6.6 | 0.2 | 2.8 | 24.6 | 0.0268 |
| 31 | 6.44 | 6.23 | 6.28 | | 5.86 | 6.04 | 6.05 | 6.2 | 0.2 | 3.4 | 32.3 | 0.0192 |
| 32 | 2.98 | 2.74 | 3.09 | | 2.58 | 3.06 | 2.97 | 2.9 | 0.2 | 6.9 | 8.5 | 0.0341 |
| 33 | 4.69 | 5.19 | 4.81 | | 5.14 | 4.85 | 5.27 | 5.0 | 0.2 | 4.8 | 25.3 | 0.0198 |
| 34 | 7.59 | 7.82 | 7.60 | | 7.46 | 7.33 | 7.74 | 7.6 | 0.2 | 2.4 | 39.9 | 0.0190 |
| 35 | 4.89 | 5.08 | 4.70 | | 5.25 | 4.90 | 4.92 | 5.0 | 0.2 | 3.8 | 16.4 | 0.0305 |
| 36 | 6.72 | 6.70 | 7.01 | | 6.81 | 6.83 | 6.77 | 6.8 | 0.1 | 1.6 | 27.3 | 0.0249 |
| 37 | 12.9 | 13.1 | 12.7 | | 13.3 | 13.3 | 13.3 | 13.1 | 0.3 | 1.9 | 39.9 | 0.0328 |
| 38 | 14.5 | 14.2 | 14.8 | | 15.0 | 15.1 | 15.4 | 14.8 | 0.4 | 2.9 | 51.9 | 0.0285 |
| 39 | 8.60 | 7.84 | 8.35 | | 7.80 | 8.15 | 7.97 | 8.1 | 0.3 | 3.8 | 46.0 | 0.0176 |
| 40 | 4.82 | 4.49 | 4.82 | | 4.30 | 4.73 | 4.72 | 4.6 | 0.2 | 4.5 | 23.2 | 0.0198 |

^a^ Values for Total 25(OH)D determined at NIST using RMPs as reported in Wise et al. [6].

Table S5. Measurements of Free 25(OH)D using the ELISA assay in 40 patient samples at Future Diagnostics compared with 25(OH)D concentration determined using a Reference Measurement Procedure

| **Sample No.** | **Free 25(OH)D (pg/mL)** | | | | | | | | | | **Total**  **25(OH)D**  **(ng/mL)^a^** | **Percent**  **Free 25(OH)D** |
| --- | --- | --- | --- | --- | --- | --- | --- | --- | --- | --- | --- | --- |
|  | **Day 1** | | | **Day 2** | | **Day 3** | |  | | |  |  |
|  | **Rep 1** | **Rep 2** | **Rep 1** | | **Rep 2** | **Rep 1** | **Rep 2** | **Mean** | **SD** | **CV** |  |  |
| 1 | 11.7 | 9.97 | 10.9 | | 9.98 | 10.8 | 9.92 | 10.55 | 0.72 | 6.8 | 61.1 | 0.0173 |
| 2 | 11.3 | 10.4 | 11.1 | | 9.26 | 11.5 | 10.3 | 10.64 | 0.83 | 7.8 | 59.8 | 0.0178 |
| 3 | 9.97 | 9.48 | 9.79 | | 8.65 | 10.0 | 9.01 | 9.48 | 0.55 | 5.8 | 56.2 | 0.0169 |
| 4 | 12.9 | 12.0 | 12.1 | | 12.6 | 13.0 | 12.3 | 12.48 | 0.42 | 3.3 | 45.1 | 0.0277 |
| 5 | 9.90 | 9.52 | 9.79 | | 8.96 | 9.59 | 9.51 | 9.55 | 0.33 | 3.4 | 42.9 | 0.0223 |
| 6 | 12.2 | 12.2 | 12.0 | | 11.5 | 12.8 | 11.8 | 12.08 | 0.44 | 3.6 | 35.1 | 0.0344 |
| 7 | 8.70 | 7.79 | 8.06 | | 7.42 | 7.93 | 8.29 | 8.03 | 0.44 | 5.4 | 29.2 | 0.0275 |
| 8 | 9.46 | 8.85 | 9.61 | | 8.32 | 9.49 | 8.78 | 9.09 | 0.51 | 5.6 | 31.1 | 0.0292 |
| 9 | 2.86 | 2.77 | 2.58 | | 2.40 | 2.61 | 2.55 | 2.63 | 0.16 | 6.2 | 6.5 | 0.0405 |
| 10 | 9.17 | 8.32 | 10.2 | | 8.62 | 9.37 | 8.89 | 9.10 | 0.66 | 7.2 | 35.8 | 0.0254 |
| 11 | 9.06 | 8.67 | 8.51 | | 8.64 | 8.57 | 8.60 | 8.68 | 0.20 | 2.3 | 35.9 | 0.0242 |
| 12 | 7.86 | 7.87 | 8.05 | | 7.77 | 8.12 | 7.99 | 7.94 | 0.13 | 1.7 | 32.5 | 0.0244 |
| 13 | 8.50 | 8.80 | 8.53 | | 8.42 | 8.78 | 8.78 | 8.64 | 0.17 | 2.0 | 30.6 | 0.0282 |
| 14 | 5.22 | 5.04 | 5.51 | | 4.62 | 5.28 | 4.78 | 5.08 | 0.33 | 6.5 | 21.6 | 0.0235 |
| 15 | 6.04 | 6.59 | 6.40 | | 6.21 | 6.31 | 6.53 | 6.35 | 0.20 | 3.2 | 29.4 | 0.0216 |
| 16 | 10.2 | 10.6 | 11.5 | | 10.2 | 10.9 | 10.5 | 10.65 | 0.49 | 4.6 | 24.9 | 0.0428 |
| 17 | 3.62 | 3.40 | 3.27 | | 3.08 | 3.48 | 3.27 | 3.35 | 0.19 | 5.6 | 13.6 | 0.0246 |
| 18 | 4.49 | 4.69 | 4.29 | | 3.91 | 4.57 | 3.98 | 4.32 | 0.32 | 7.4 | 17.3 | 0.0250 |
| 19 | 16.1 | 16.5 | 14.5 | | 14.9 | 15.4 | 15.6 | 15.50 | 0.74 | 4.8 | 50.2 | 0.0309 |
| 20 | 4.58 | 4.74 | 4.32 | | 4.39 | 4.75 | 4.52 | 4.55 | 0.18 | 3.9 | 25.5 | 0.0178 |
| 21 | 7.04 | 6.94 | 6.33 | | 6.31 | 6.79 | 6.94 | 6.73 | 0.32 | 4.8 | 32.5 | 0.0207 |
| 22 | 3.83 | 3.72 | 2.98 | | 3.27 | 3.32 | 3.43 | 3.43 | 0.31 | 9.1 | 12.3 | 0.0279 |
| 23 | 4.07 | 4.44 | 3.42 | | 4.00 | 3.77 | 4.08 | 3.96 | 0.34 | 8.6 | 17.2 | 0.0230 |
| 24 | 3.45 | 3.49 | 2.81 | | 3.33 | 3.21 | 3.37 | 3.28 | 0.25 | 7.6 | 11.6 | 0.0283 |
| 25 | 5.13 | 4.96 | 4.72 | | 4.23 | 4.96 | 4.82 | 4.80 | 0.31 | 6.5 | 30.1 | 0.0159 |
| 26 | 3.84 | 4.10 | 3.51 | | 3.95 | 3.82 | 3.82 | 3.84 | 0.19 | 5.1 | 12.0 | 0.0320 |
| 27 | 9.06 | 8.75 | 8.49 | | 8.23 | 8.42 | 8.60 | 8.59 | 0.29 | 3.4 | 33.8 | 0.0254 |
| 28 | 4.69 | 4.56 | 4.43 | | 4.53 | 4.57 | 4.60 | 4.56 | 0.09 | 1.9 | 21.3 | 0.0214 |
| 29 | 5.51 | 5.21 | 4.98 | | 5.33 | 4.85 | 5.46 | 5.22 | 0.26 | 5.1 | 20.2 | 0.0258 |
| 30 | 6.87 | 7.21 | 6.39 | | 6.30 | 6.40 | 6.31 | 6.58 | 0.37 | 5.7 | 24.6 | 0.0267 |
| 31 | 6.20 | 6.11 | 6.06 | | 5.76 | 6.15 | 6.08 | 6.06 | 0.16 | 2.6 | 32.3 | 0.0188 |
| 32 | 2.77 | 3.10 | 2.24 | | 2.63 | 2.81 | 2.96 | 2.75 | 0.30 | 10.8 | 8.5 | 0.0324 |
| 33 | 4.80 | 4.82 | 4.89 | | 4.75 | 4.93 | 4.94 | 4.86 | 0.08 | 1.6 | 25.3 | 0.0192 |
| 34 | 7.31 | 6.90 | 7.69 | | 7.33 | 7.48 | 7.19 | 7.32 | 0.27 | 3.6 | 39.9 | 0.0183 |
| 35 | 4.83 | 4.93 | 4.69 | | 4.47 | 4.59 | 4.83 | 4.72 | 0.17 | 3.6 | 16.4 | 0.0288 |
| 36 | 6.16 | 5.96 | 6.36 | | 6.68 | 6.58 | 6.35 | 6.35 | 0.26 | 4.2 | 27.3 | 0.0233 |
| 37 | 12.6 | 11.6 | 12.7 | | 11.6 | 11.7 | 12.2 | 12.07 | 0.50 | 4.2 | 39.9 | 0.0303 |
| 38 | 14.5 | 13.7 | 14.1 | | 14.3 | 14.3 | 13.5 | 14.07 | 0.39 | 2.8 | 51.9 | 0.0271 |
| 39 | 7.46 | 7.75 | 7.38 | | 7.86 | 7.79 | 7.96 | 7.70 | 0.23 | 3.0 | 46.0 | 0.0167 |
| 40 | 4.52 | 4.36 | 4.43 | | 4.63 | 4.66 | 4.63 | 4.54 | 0.12 | 2.7 | 23.2 | 0.0173 |

^a^ Values for Total 25(OH)D determined at NIST using RMPs as reported in Wise et al. [6].

Figure S1. Ordinary least squares linear regression for free 25(OH)D and total 25(OH)D in 40 single-donor patient samples. Black circles are the single-donor samples and the solid red line is the regression line. Red dashed line is the 95% confidence interval for the regression line. Free 25(OH)D measurements performed at Future Diagnostics.


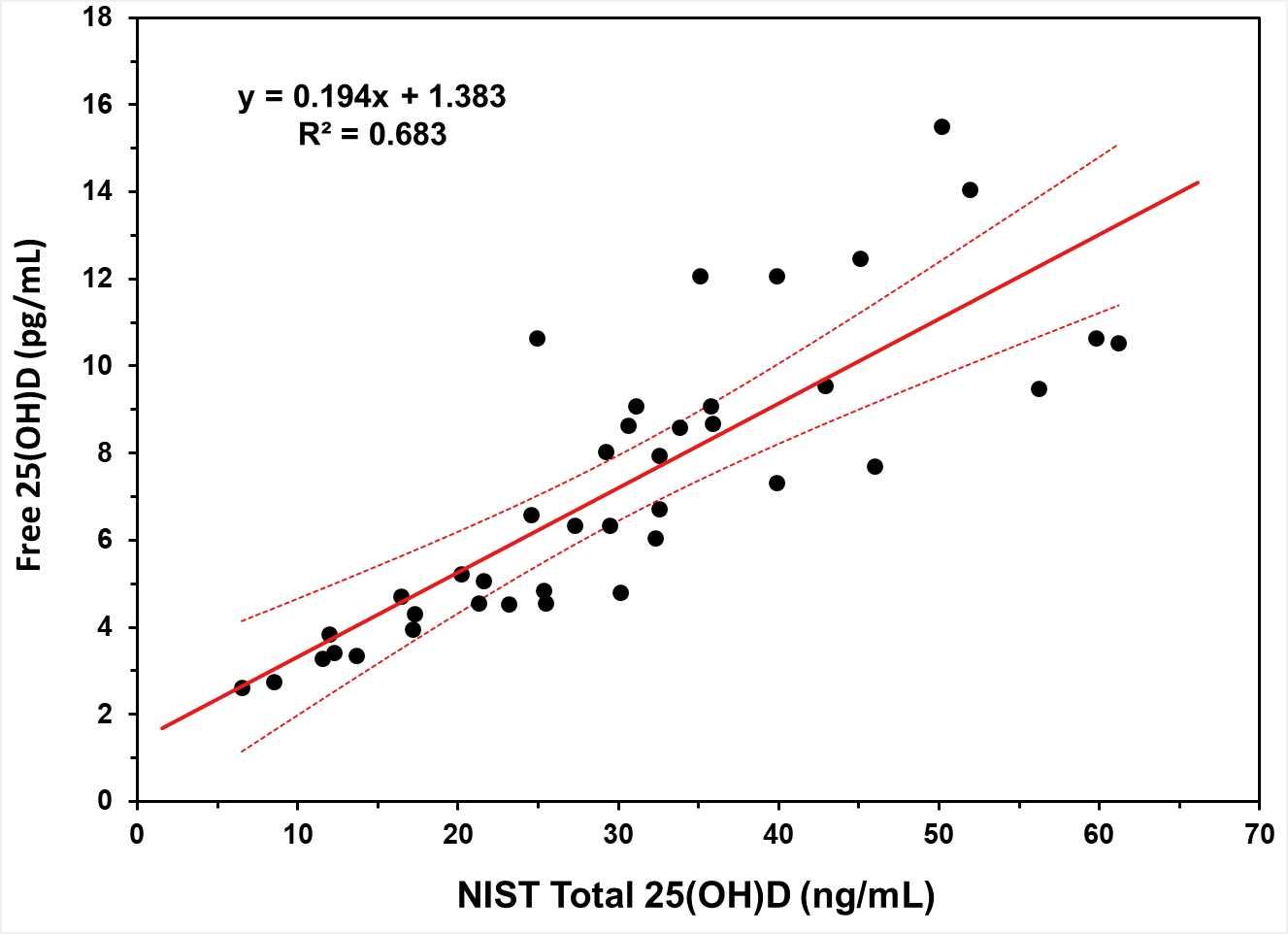


Figure S2. Distribution of free 25-hydroxyvitamin D (pg/mL) measurements by laboratory for NIST SRM 972a – Vitamin D Metabolites in Frozen Human Serum (Level 1). Blue diamonds are the individual measurements. Black bar is the mean value of the 12 measurements.


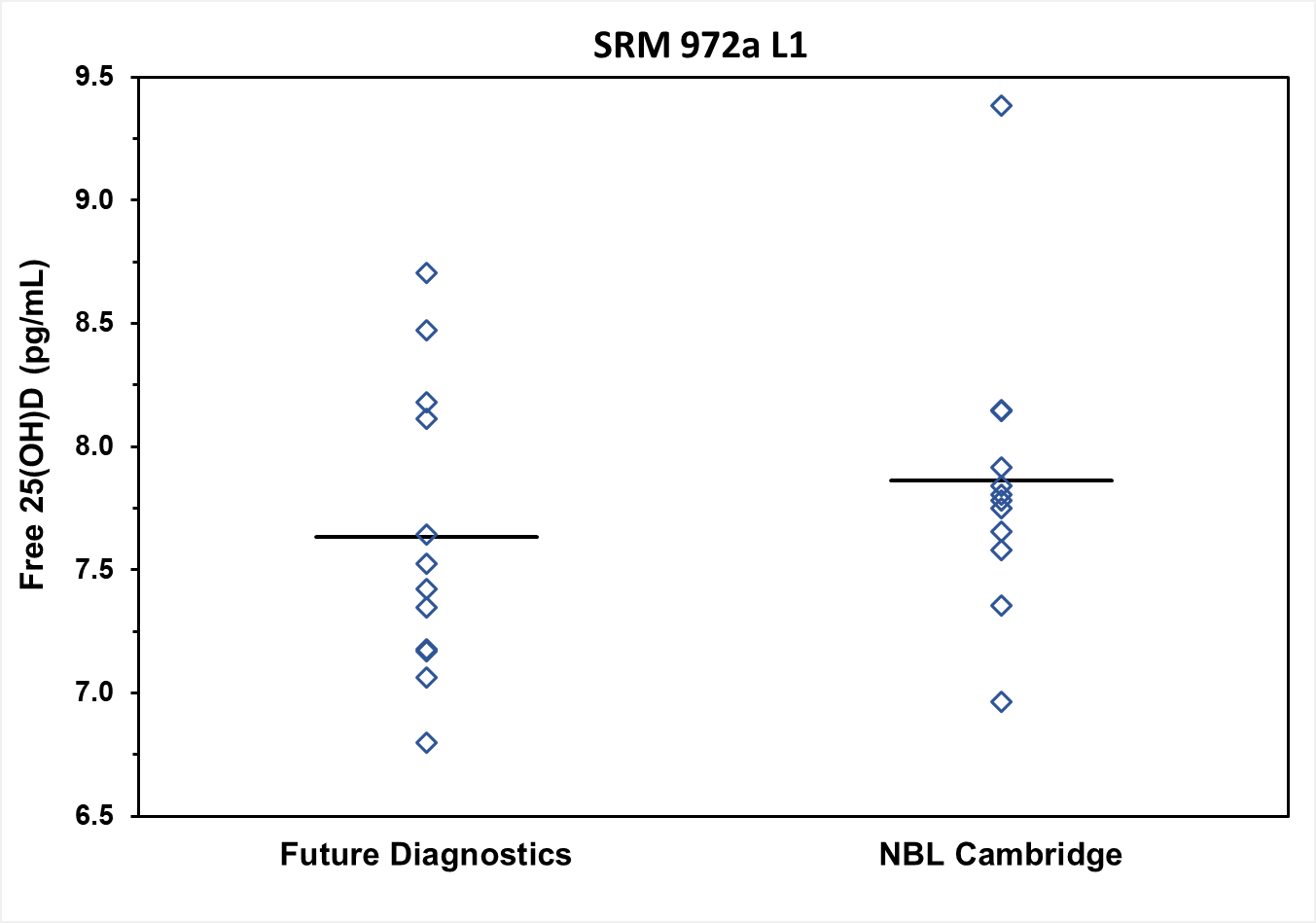


Figure S3. Distribution of free 25-hydroxyvitamin D (pg/mL) measurements by laboratory for NIST SRM 972a – Vitamin D Metabolites in Frozen Human Serum (Level 2). Blue diamonds are the individual measurements. Black bar is the mean value of the 12 measurements.


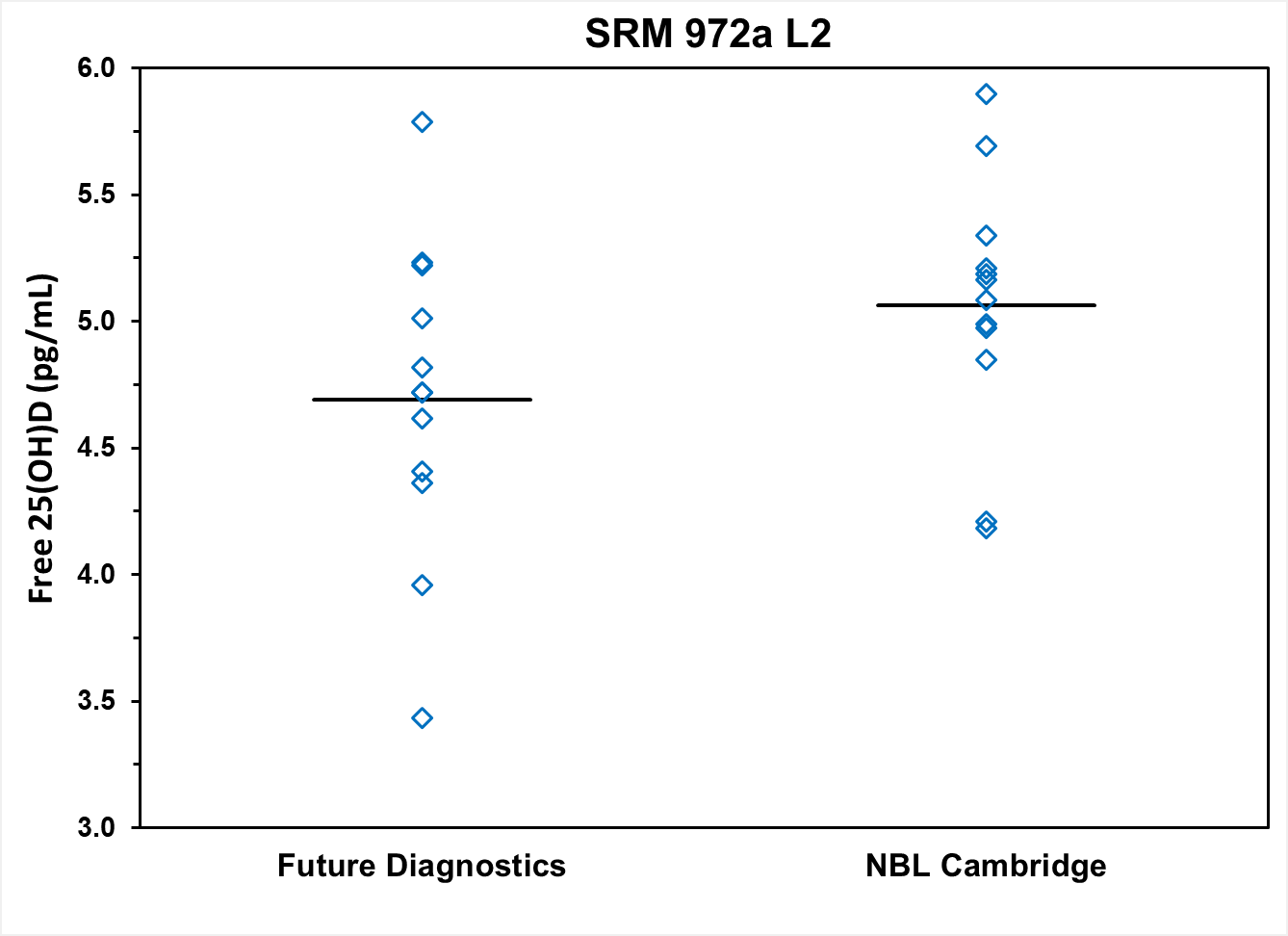


Figure S4. Distribution of free 25-hydroxyvitamin D (pg/mL) measurements by laboratory for NIST SRM 972a – Vitamin D Metabolites in Frozen Human Serum (Level 4). Blue diamonds are the individual measurements. Black bar is the mean value of the 12 measurements.


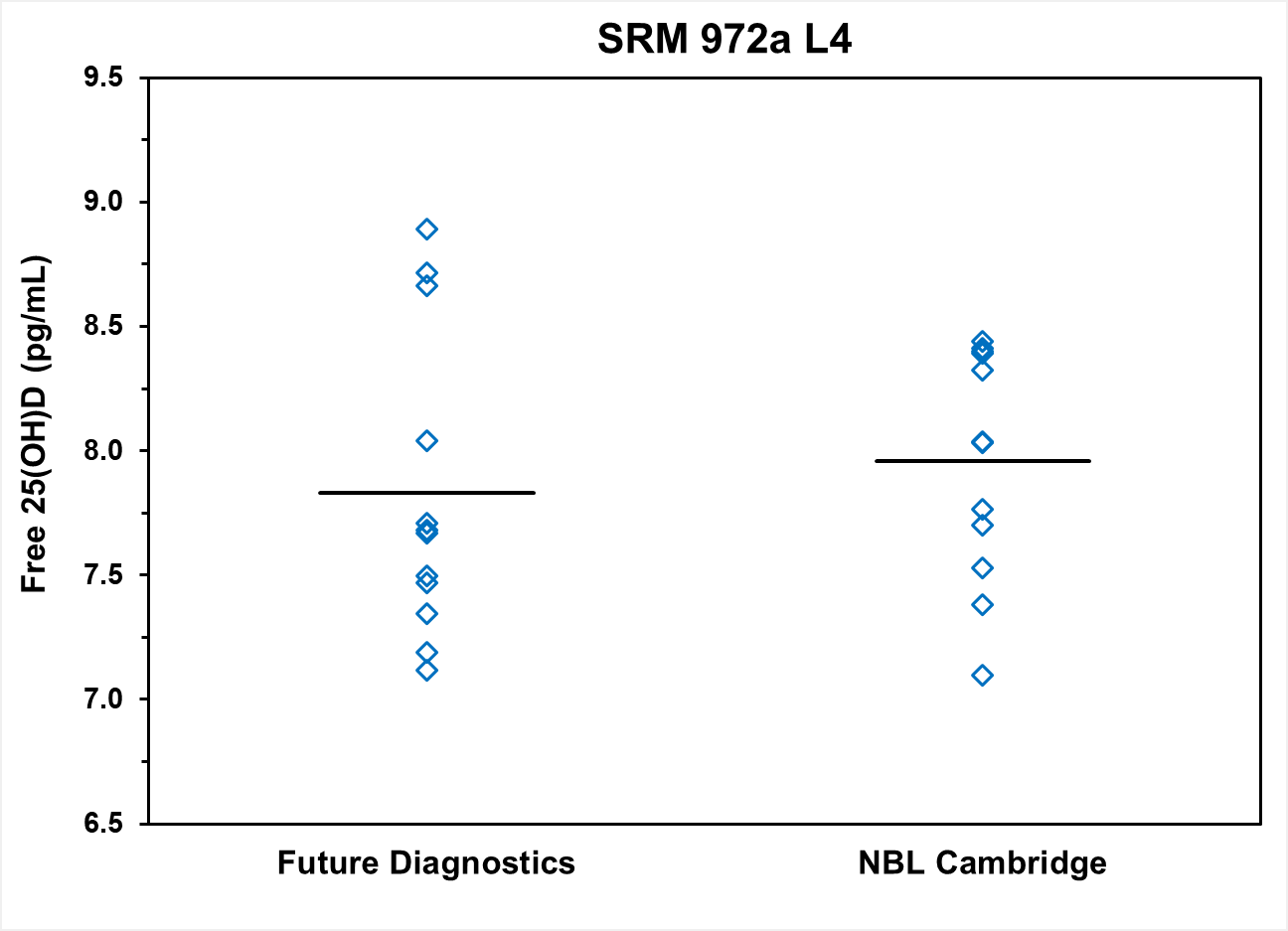


Figure S5. Distribution of free 25-hydroxyvitamin D (pg/mL) measurements by laboratory for NIST SRM 2973 – Vitamin D Metabolites in Frozen Human Serum (High Level). Blue diamonds are the individual measurements. Black bar is the mean value of the 12 measurements.


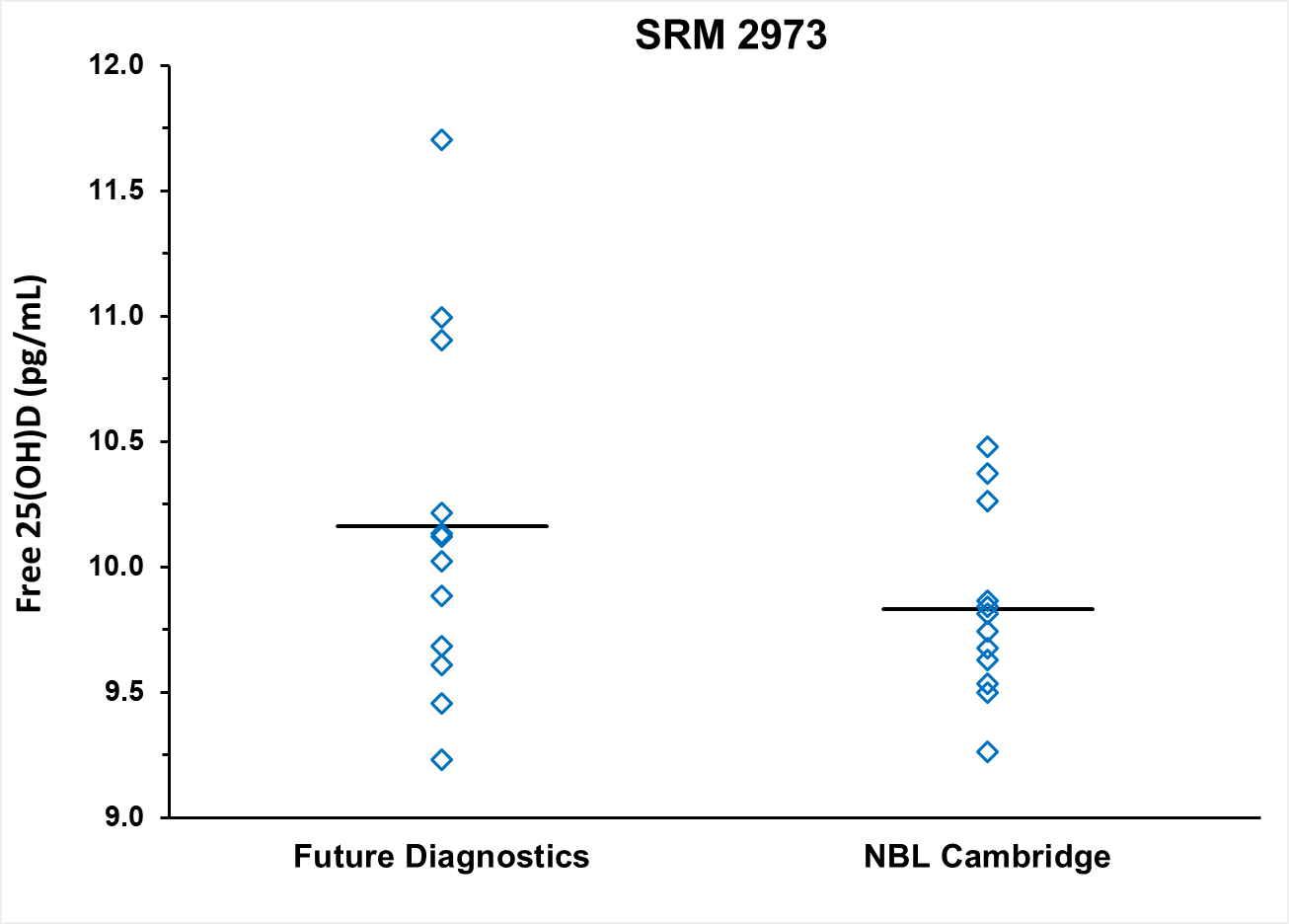


Figure S6. Distribution of free 25-hydroxyvitamin D (pg/mL) measurements by laboratory for NIST SRM 1949 – Frozen Human Prenatal Serum (Non-Pregnant). Blue diamonds are the individual measurements. Black bar is the mean value of the 12 measurements.


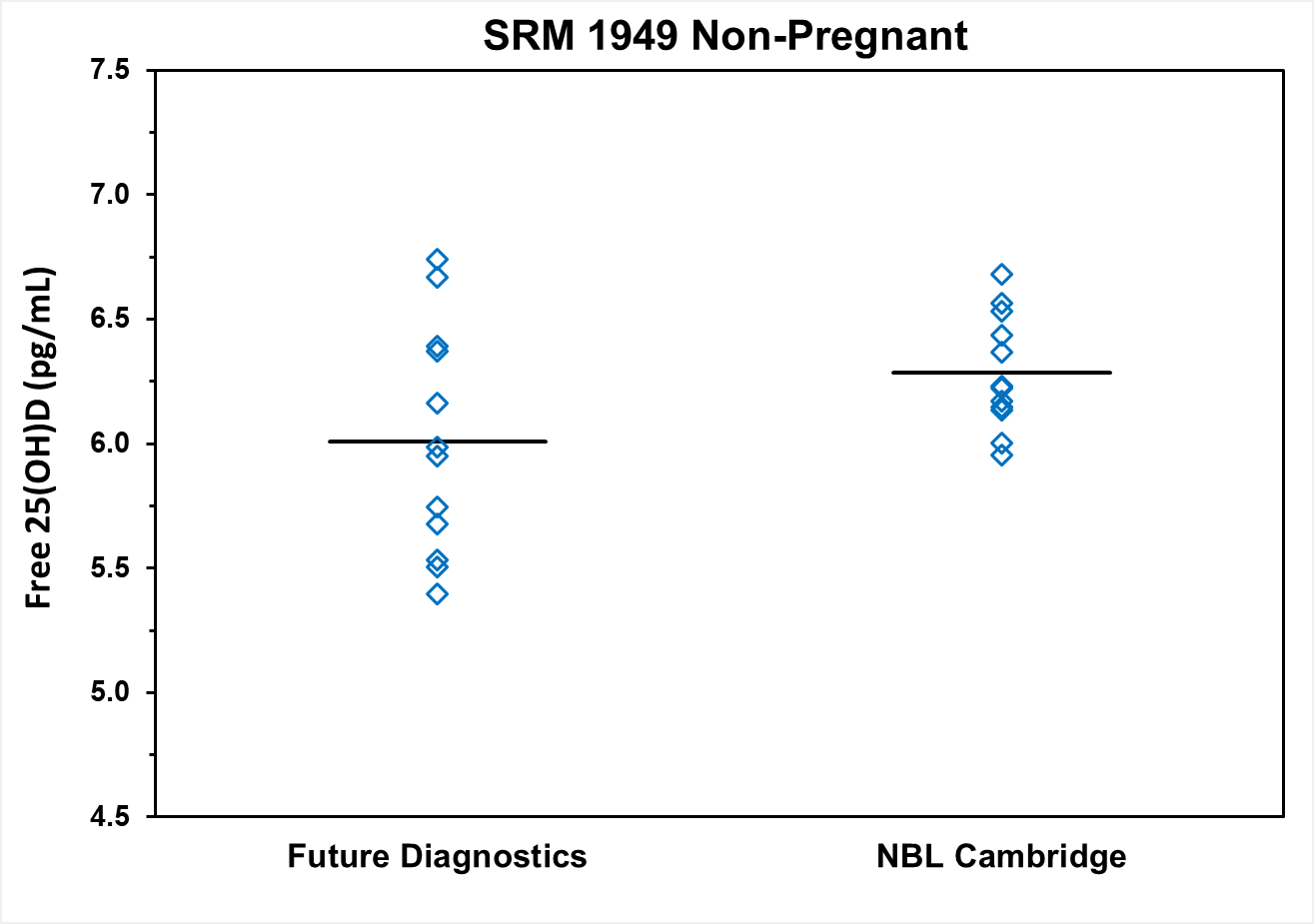


Figure S7. Distribution of free 25-hydroxyvitamin D (pg/mL) measurements by laboratory for NIST SRM 1949 – Frozen Human Prenatal Serum (First Trimester). Blue diamonds are the individual measurements. Black bar is the mean value of the 12 measurements.

**
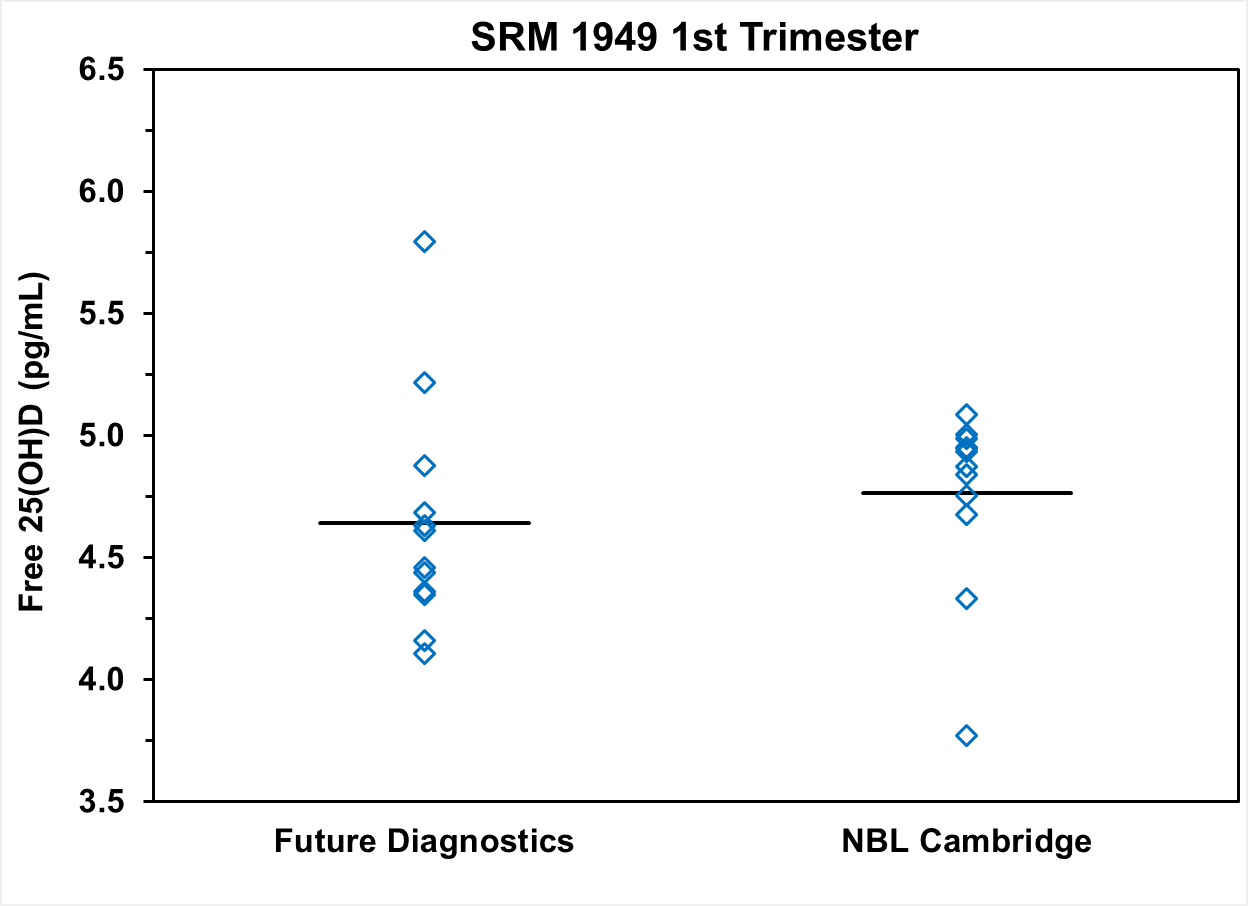
**

Figure S8. Distribution of free 25-hydroxyvitamin D (pg/mL) measurements by laboratory for NIST SRM 1949 – Frozen Human Prenatal Serum (Second Trimester). Blue diamonds are the individual measurements. Black bar is the mean value of the 12 measurements.


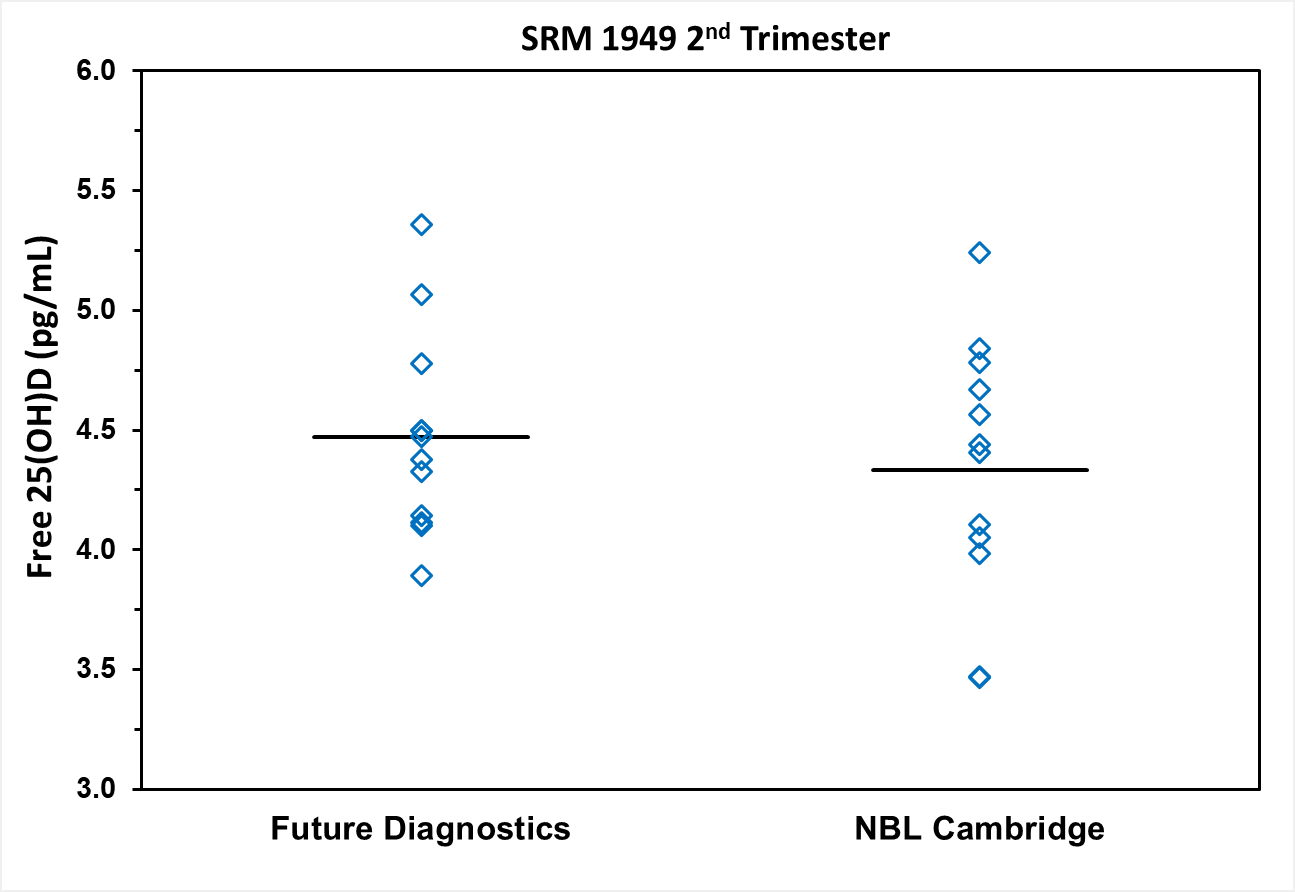


Figure S9. Distribution of free 25-hydroxyvitamin D (pg/mL) measurements by laboratory for NIST SRM 1949 – Frozen Human Prenatal Serum (Third Trimester). Blue diamonds are the individual measurements. Black bar is the mean value of the 12 measurements.


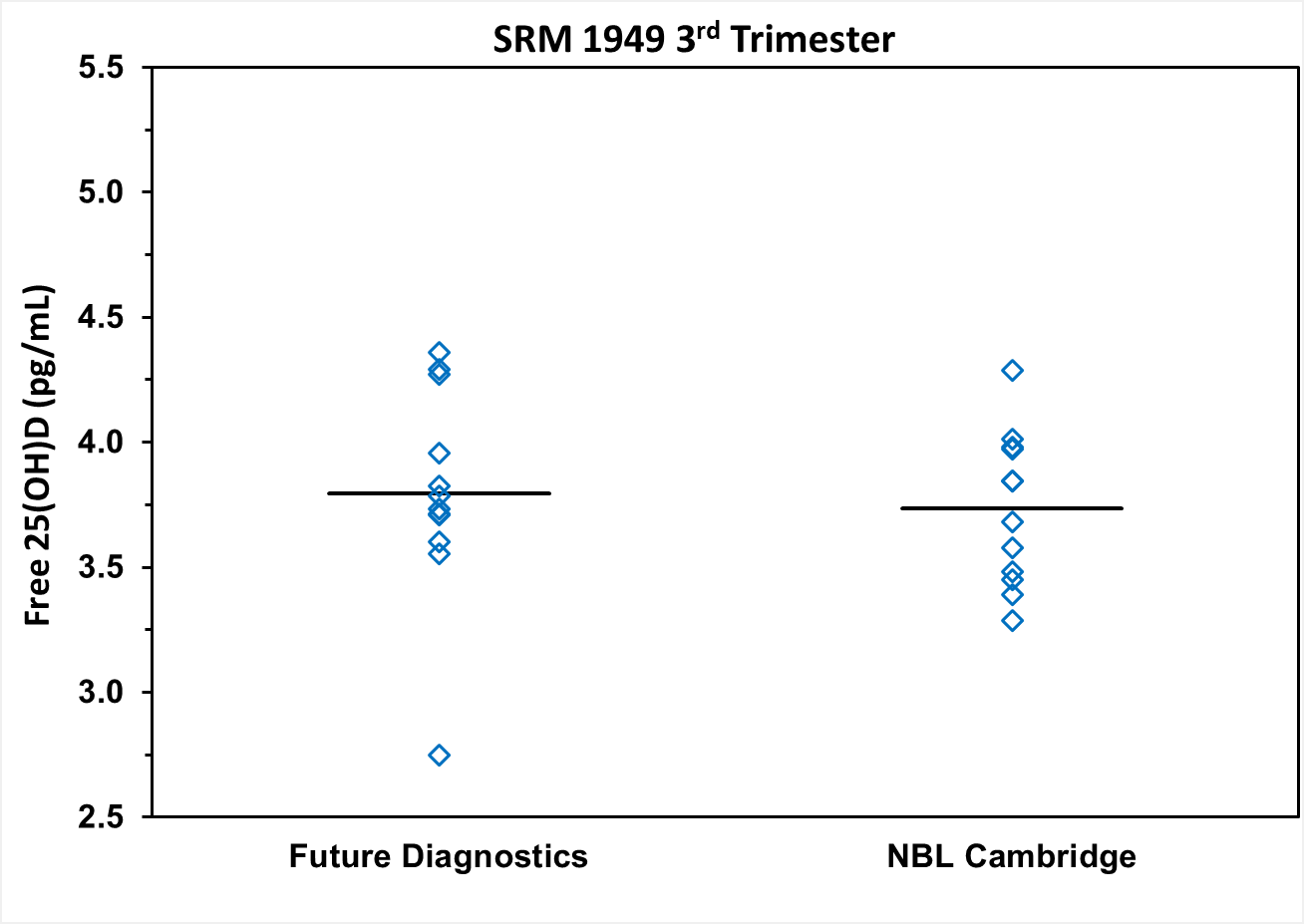


Figure S10. Standardized residual plot for ordinary least squares regression model of the means of directly measured free 25-hydroxyvitamin D concentrations for Future Diagnostic and NBL Cambridge.


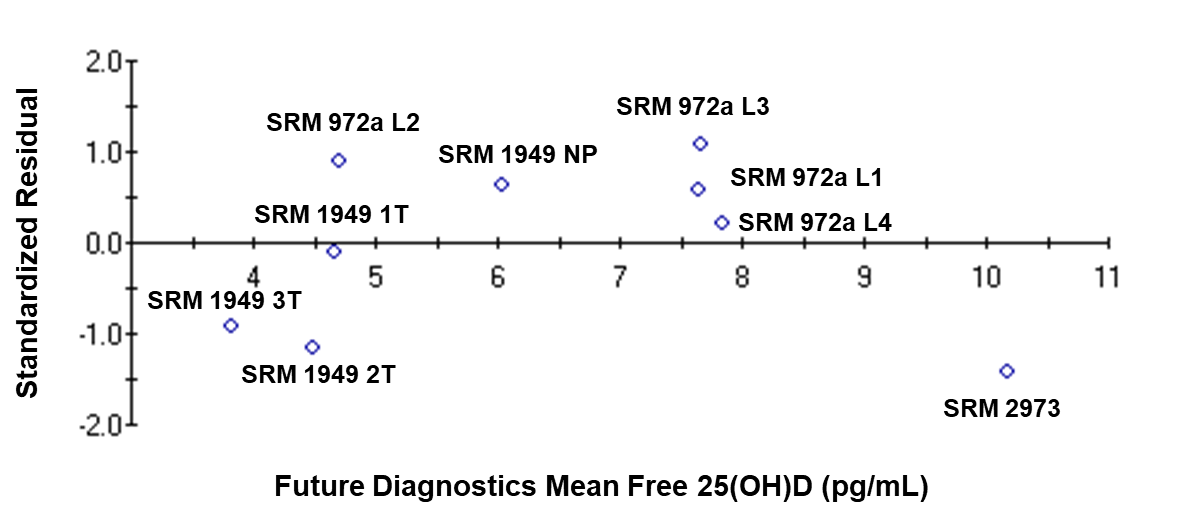

Supplement: Supplementary file 1 — Supplementary file1 (DOCX 297 KB) [file 216_2022_4313_MOESM1_ESM.docx]
